# Review of deep learning: concepts, CNN architectures, challenges, applications, future directions

**DOI:** 10.1186/s40537-021-00444-8

**Published:** 2021-03-31

**Authors:** Laith Alzubaidi, Jinglan Zhang, Amjad J. Humaidi, Ayad Al-Dujaili, Ye Duan, Omran Al-Shamma, J. Santamaría, Mohammed A. Fadhel, Muthana Al-Amidie, Laith Farhan

**Affiliations:** 1 School of Computer Science, Queensland University of Technology, Brisbane, QLD 4000 Australia; 2 Control and Systems Engineering Department, University of Technology, Baghdad, 10001 Iraq; 3 Electrical Engineering Technical College, Middle Technical University, Baghdad, 10001 Iraq; 4 Faculty of Electrical Engineering & Computer Science, University of Missouri, Columbia, MO 65211 USA; 5 AlNidhal Campus, University of Information Technology & Communications, Baghdad, 10001 Iraq; 6 Department of Computer Science, University of Jaén, 23071 Jaén, Spain; 7 College of Computer Science and Information Technology, University of Sumer, Thi Qar, 64005 Iraq; 8 School of Engineering, Manchester Metropolitan University, Manchester, M1 5GD UK

**Keywords:** Deep learning, Machine learning, Convolution neural network (CNN), Deep neural network architectures, Deep learning applications, Image classification, Transfer learning, Medical image analysis, Supervised learning, FPGA, GPU

## Abstract

In the last few years, the deep learning (DL) computing paradigm has been deemed the Gold Standard in the machine learning (ML) community. Moreover, it has gradually become the most widely used computational approach in the field of ML, thus achieving outstanding results on several complex cognitive tasks, matching or even beating those provided by human performance. One of the benefits of DL is the ability to learn massive amounts of data. The DL field has grown fast in the last few years and it has been extensively used to successfully address a wide range of traditional applications. More importantly, DL has outperformed well-known ML techniques in many domains, e.g., cybersecurity, natural language processing, bioinformatics, robotics and control, and medical information processing, among many others. Despite it has been contributed several works reviewing the State-of-the-Art on DL, all of them only tackled one aspect of the DL, which leads to an overall lack of knowledge about it. Therefore, in this contribution, we propose using a more holistic approach in order to provide a more suitable starting point from which to develop a full understanding of DL. Specifically, this review attempts to provide a more comprehensive survey of the most important aspects of DL and including those enhancements recently added to the field. In particular, this paper outlines the importance of DL, presents the types of DL techniques and networks. It then presents convolutional neural networks (CNNs) which the most utilized DL network type and describes the development of CNNs architectures together with their main features, e.g., starting with the AlexNet network and closing with the High-Resolution network (HR.Net). Finally, we further present the challenges and suggested solutions to help researchers understand the existing research gaps. It is followed by a list of the major DL applications. Computational tools including FPGA, GPU, and CPU are summarized along with a description of their influence on DL. The paper ends with the evolution matrix, benchmark datasets, and summary and conclusion.

## Introduction

Recently, machine learning (ML) has become very widespread in research and has been incorporated in a variety of applications, including text mining, spam detection, video recommendation, image classification, and multimedia concept retrieval [1–6]. Among the different ML algorithms, deep learning (DL) is very commonly employed in these applications [7–9]. Another name for DL is representation learning (RL). The continuing appearance of novel studies in the fields of deep and distributed learning is due to both the unpredictable growth in the ability to obtain data and the amazing progress made in the hardware technologies, e.g. High Performance Computing (HPC) [10].

DL is derived from the conventional neural network but considerably outperforms its predecessors. Moreover, DL employs transformations and graph technologies simultaneously in order to build up multi-layer learning models. The most recently developed DL techniques have obtained good outstanding performance across a variety of applications, including audio and speech processing, visual data processing, natural language processing (NLP), among others [11–14].

Usually, the effectiveness of an ML algorithm is highly dependent on the integrity of the input-data representation. It has been shown that a suitable data representation provides an improved performance when compared to a poor data representation. Thus, a significant research trend in ML for many years has been feature engineering, which has informed numerous research studies. This approach aims at constructing features from raw data. In addition, it is extremely field-specific and frequently requires sizable human effort. For instance, several types of features were introduced and compared in the computer vision context, such as, histogram of oriented gradients (HOG) [15], scale-invariant feature transform (SIFT) [16], and bag of words (BoW) [17]. As soon as a novel feature is introduced and is found to perform well, it becomes a new research direction that is pursued over multiple decades.

Relatively speaking, feature extraction is achieved in an automatic way throughout the DL algorithms. This encourages researchers to extract discriminative features using the smallest possible amount of human effort and field knowledge [18]. These algorithms have a multi-layer data representation architecture, in which the first layers extract the low-level features while the last layers extract the high-level features. Note that artificial intelligence (AI) originally inspired this type of architecture, which simulates the process that occurs in core sensorial regions within the human brain. Using different scenes, the human brain can automatically extract data representation. More specifically, the output of this process is the classified objects, while the received scene information represents the input. This process simulates the working methodology of the human brain. Thus, it emphasizes the main benefit of DL.

In the field of ML, DL, due to its considerable success, is currently one of the most prominent research trends. In this paper, an overview of DL is presented that adopts various perspectives such as the main concepts, architectures, challenges, applications, computational tools and evolution matrix. Convolutional neural network (CNN) is one of the most popular and used of DL networks [19, 20]. Because of CNN, DL is very popular nowadays. The main advantage of CNN compared to its predecessors is that it automatically detects the significant features without any human supervision which made it the most used. Therefore, we have dug in deep with CNN by presenting the main components of it. Furthermore, we have elaborated in detail the most common CNN architectures, starting with the AlexNet network and ending with the High-Resolution network (HR.Net).

Several published DL review papers have been presented in the last few years. However, all of them have only been addressed one side focusing on one application or topic such as the review of CNN architectures [21], DL for classification of plant diseases [22], DL for object detection [23], DL applications in medical image analysis [24], and etc. Although these reviews present good topics, they do not provide a full understanding of DL topics such as concepts, detailed research gaps, computational tools, and DL applications. First, It is required to understand DL aspects including concepts, challenges, and applications then going deep in the applications. To achieve that, it requires extensive time and a large number of research papers to learn about DL including research gaps and applications. Therefore, we propose a deep review of DL to provide a more suitable starting point from which to develop a full understanding of DL from one review paper. The motivation behinds our review was to cover the most important aspect of DL including open challenges, applications, and computational tools perspective. Furthermore, our review can be the first step towards other DL topics.

The main aim of this review is to present the most important aspects of DL to make it easy for researchers and students to have a clear image of DL from single review paper. This review will further advance DL research by helping people discover more about recent developments in the field. Researchers would be allowed to decide the more suitable direction of work to be taken in order to provide more accurate alternatives to the field. Our contributions are outlined as follows:

- This is the first review that almost provides a deep survey of the most important aspects of deep learning. This review helps researchers and students to have a good understanding from one paper.
- We explain CNN in deep which the most popular deep learning algorithm by describing the concepts, theory, and state-of-the-art architectures.
- We review current challenges (limitations) of Deep Learning including lack of training data, Imbalanced Data, Interpretability of data, Uncertainty scaling, Catastrophic forgetting, Model compression, Overfitting, Vanishing gradient problem, Exploding Gradient Problem, and Underspecification. We additionally discuss the proposed solutions tackling these issues.
- We provide an exhaustive list of medical imaging applications with deep learning by categorizing them based on the tasks by starting with classification and ending with registration.
- We discuss the computational approaches (CPU, GPU, FPGA) by comparing the influence of each tool on deep learning algorithms.

The rest of the paper is organized as follows: “Survey methodology” section describes The survey methodology. “Background” section presents the background. “Classification of DL approaches” section defines the classification of DL approaches. “Types of DL networks” section displays types of DL networks. “CNN architectures” section shows CNN Architectures. “Challenges (limitations) of deep learning and alternate solutions” section details the challenges of DL and alternate solutions. “Applications of deep learning” section outlines the applications of DL. “Computational approaches” section explains the influence of computational approaches (CPU, GPU, FPGA) on DL. “Evaluation metrics” section presents the evaluation metrics. “Frameworks and datasets” section lists frameworks and datasets. “Summary and conclusion” section presents the summary and conclusion.

## Survey methodology

**Fig. 1:**
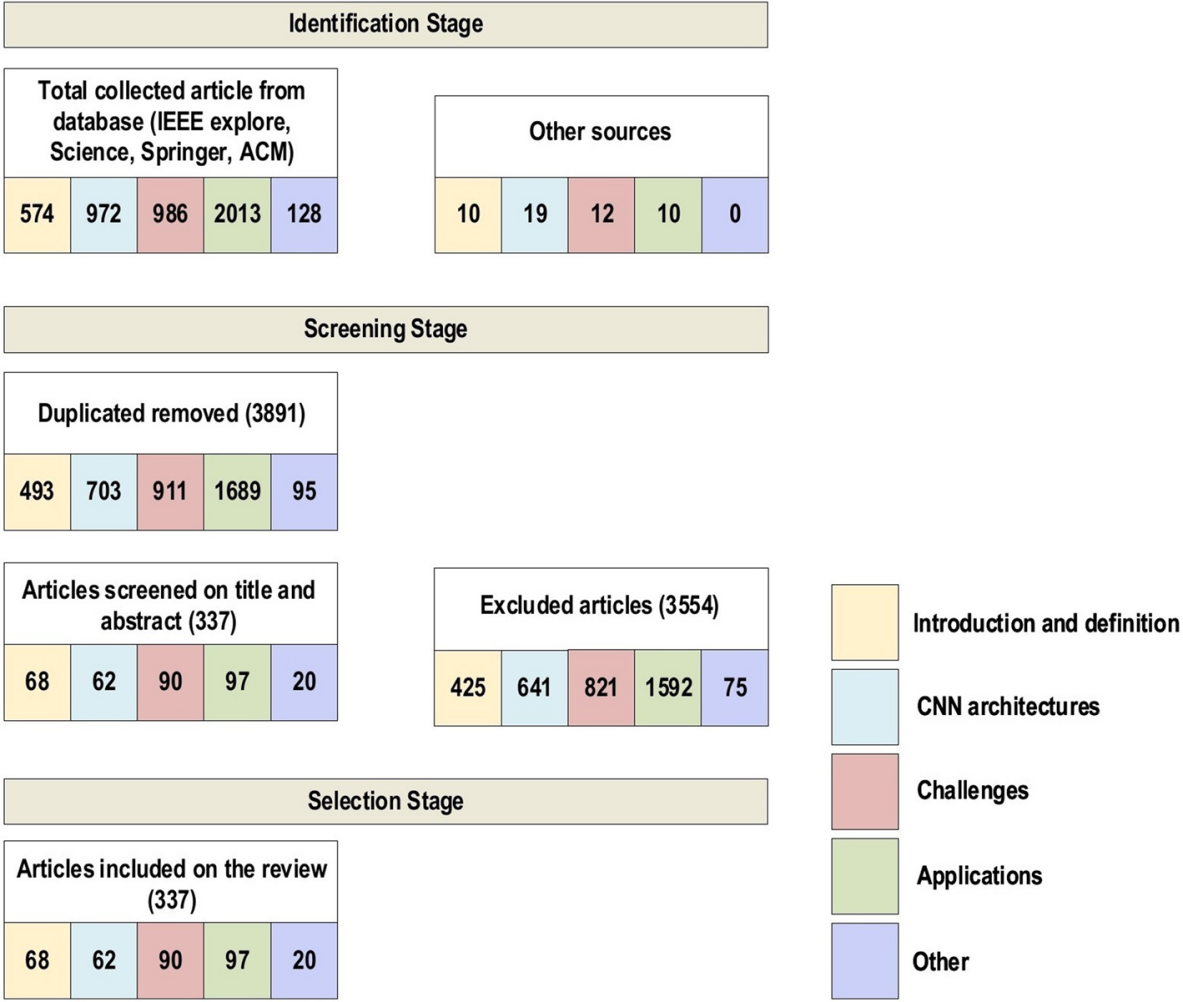
Search framework

We have reviewed the significant research papers in the field published during 2010–2020, mainly from the years of 2020 and 2019 with some papers from 2021. The main focus was papers from the most reputed publishers such as IEEE, Elsevier, MDPI, Nature, ACM, and Springer. Some papers have been selected from ArXiv. We have reviewed more than 300 papers on various DL topics. There are 108 papers from the year 2020, 76 papers from the year 2019, and 48 papers from the year 2018. This indicates that this review focused on the latest publications in the field of DL. The selected papers were analyzed and reviewed to (1) list and define the DL approaches and network types, (2) list and explain CNN architectures, (3) present the challenges of DL and suggest the alternate solutions, (4) assess the applications of DL, (5) assess computational approaches. The most keywords used for search criteria for this review paper are (“Deep Learning”), (“Machine Learning”), (“Convolution Neural Network”), (“Deep Learning” AND “Architectures”), ((“Deep Learning”) AND (“Image”) AND (“detection” OR “classification” OR “segmentation” OR “Localization”)), (“Deep Learning” AND “detection” OR “classification” OR “segmentation” OR “Localization”), (“Deep Learning” AND “CPU” OR “GPU” OR “FPGA”), (“Deep Learning” AND “Transfer Learning”), (“Deep Learning” AND “Imbalanced Data”), (“Deep Learning” AND “Interpretability of data”), (“Deep Learning” AND “Overfitting”), (“Deep Learning” AND “Underspecification”). Figure 1 shows our search structure of the survey paper. Table 1 presents the details of some of the journals that have been cited in this review paper.

**Table 1:** Some of the journals have been cited in this review paper

Journal	IF 2019	CiteScore 2019	Publisher	Journal homepage
Pattern Recognition	7.196	13.1	Elsevier	https://www.journals.elsevier.com/pattern-recognition
Pattern Recognition Letter	3.255	6.3	Elsevier	https://www.journals.elsevier.com/pattern-recognition-letters
Artificial Intelligence Review	5.747	9.1	Springer	https://www.springer.com/journal/10462?referer=www.springeronline.com
Expert Systems with Applications	5.452	11	Elsevier	https://www.sciencedirect.com/journal/expert-systems-with-applications
Neurocomputing	4.438	9.5	Elsevier	https://www.journals.elsevier.com/neurocomputing
Nature Medicine	36.130	45.9	Nature	https://www.nature.com/nm/
Nature	42.779	51	Nature	https://www.nature.com/
Journal of Big Data	–	6.1	Springer	https://journalofbigdata.springeropen.com/
Multimedia Tools and Applications	2.313	3.7	Springer	https://www.springer.com/journal/11042
Computer Methods and Programs in Biomedicine	3.632	7.5	Elsevier	https://www.journals.elsevier.com/computer-methods-and-programs-in-biomedicine
Machine Learning	2.672	5.0	Springer	https://www.springer.com/journal/10994
Machine Vision and Applications	1.605	4.2	Springer	https://www.springer.com/journal/138
Medical Image Analysis	11.148	17.2	Elsevier	https://www.sciencedirect.com/journal/medical-image-analysis
IEEE Access	3.745	3.9	IEEE	https://ieeexplore.ieee.org/xpl/RecentIssue.jsp?punumber=6287639
IEEE Transactions on Knowledge and Data Engineering	4.935	12.0	IEEE	https://ieeexplore.ieee.org/xpl/RecentIssue.jsp?punumber=69
Nature Communications	12.121	18.1	Nature	https://www.nature.com/ncomms/
IEEE Transactions on Intelligent Transportation Systems	6.319	12.7	IEEE	https://ieeexplore.ieee.org/xpl/RecentIssue.jsp?punumber=6979
Methods	3.812	8.0	Elsevier	https://www.journals.elsevier.com/methods
ACM Journal on Emerging Technologies in Computing Systems	1.652	4.3	ACM	https://dl.acm.org/journal/jetc
ACM Computing Surveys	6.319	12.7	ACM	https://dl.acm.org/journal/csur
Applied Soft Computing	5.472	10.2	Elsevier	https://www.journals.elsevier.com/applied-soft-computing
Electronics	2.412	1.9	MDPI	https://www.mdpi.com/journal/electronics
Applied Sciences	2.474	2.4	MDPI	https://www.mdpi.com/journal/applsci
IEEE Transactions on Industrial Informatics	9.112	13.9	IEEE	https://ieeexplore.ieee.org/xpl/RecentIssue.jsp?punumber=9424

## Background

This section will present a background of DL. We begin with a quick introduction to DL, followed by the difference between DL and ML. We then show the situations that require DL. Finally, we present the reasons for applying DL.

**Fig. 2:**
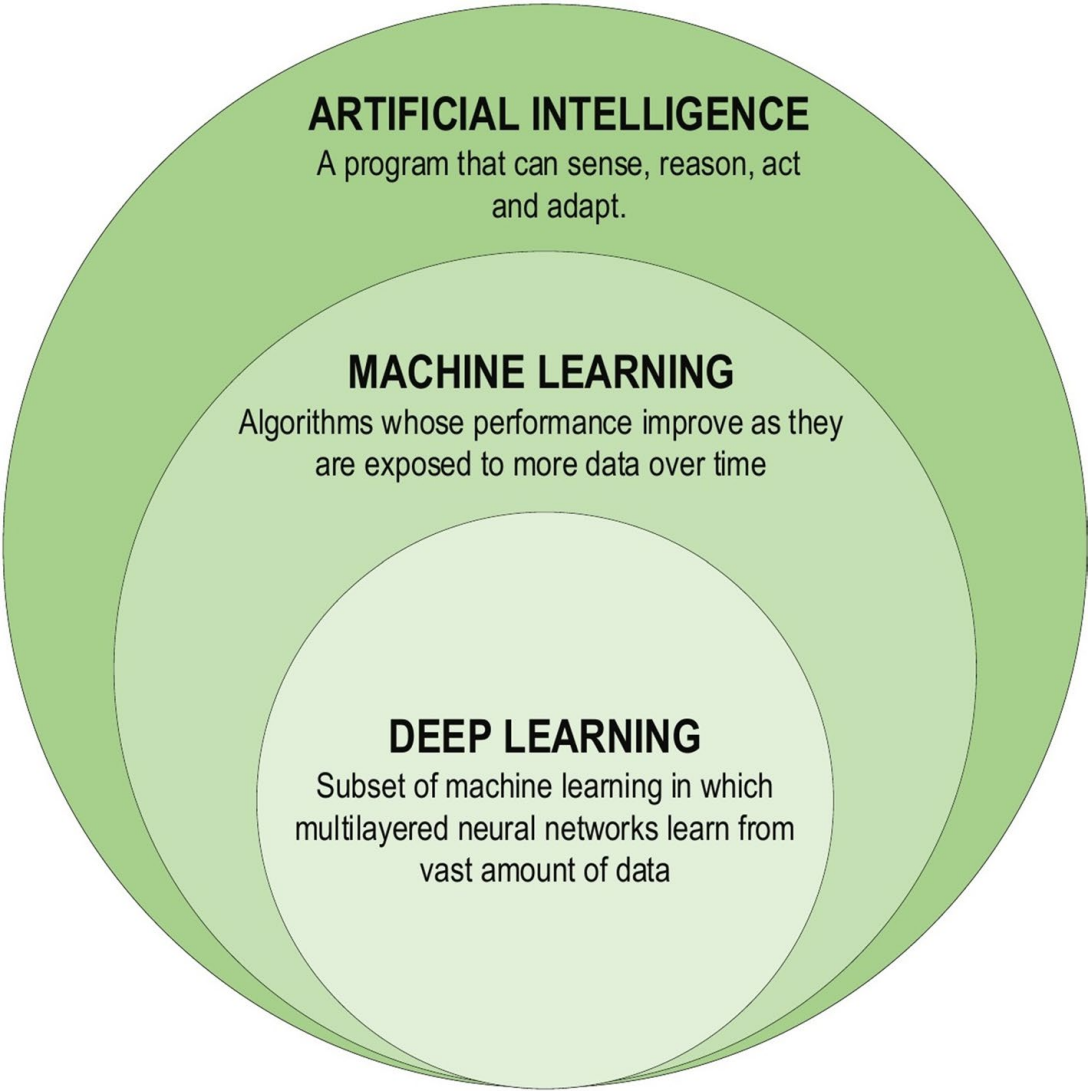
Deep learning family

DL, a subset of ML (Fig. 2), is inspired by the information processing patterns found in the human brain. DL does not require any human-designed rules to operate; rather, it uses a large amount of data to map the given input to specific labels. DL is designed using numerous layers of algorithms (artificial neural networks, or ANNs), each of which provides a different interpretation of the data that has been fed to them [18, 25].

**Fig. 3:**
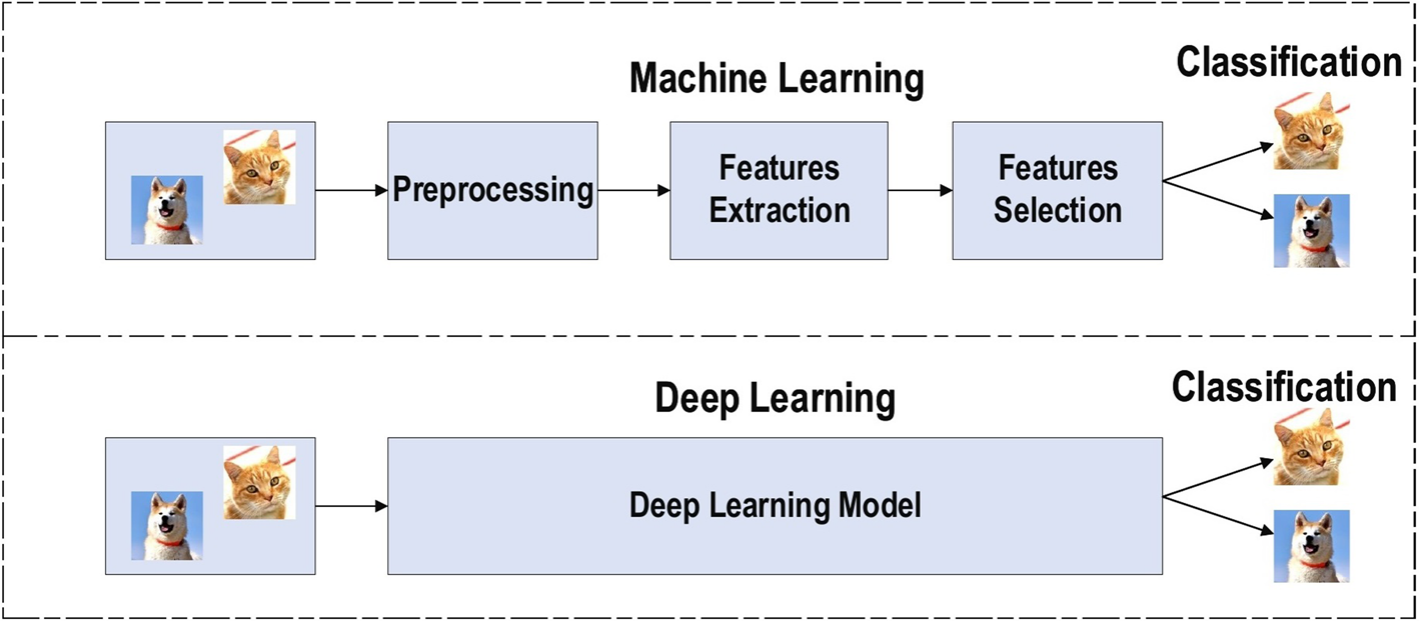
The difference between deep learning and traditional machine learning

**Fig. 4:**
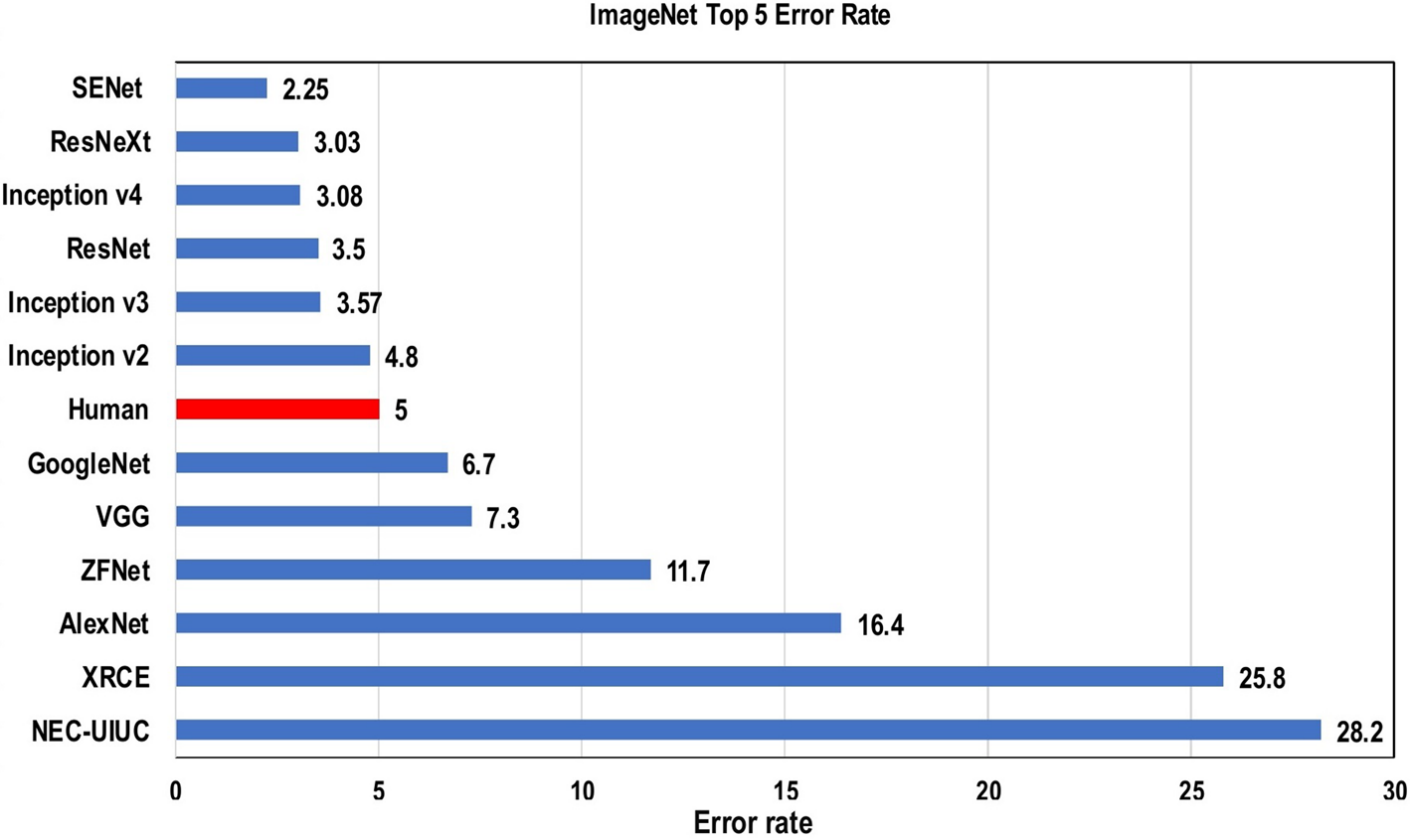
Deep learning performance compared to human

Achieving the classification task using conventional ML techniques requires several sequential steps, specifically pre-processing, feature extraction, wise feature selection, learning, and classification. Furthermore, feature selection has a great impact on the performance of ML techniques. Biased feature selection may lead to incorrect discrimination between classes. Conversely, DL has the ability to automate the learning of feature sets for several tasks, unlike conventional ML methods [18, 26]. DL enables learning and classification to be achieved in a single shot (Fig. 3). DL has become an incredibly popular type of ML algorithm in recent years due to the huge growth and evolution of the field of big data [27, 28]. It is still in continuous development regarding novel performance for several ML tasks [22, 29–31] and has simplified the improvement of many learning fields [32, 33], such as image super-resolution [34], object detection [35, 36], and image recognition [30, 37]. Recently, DL performance has come to exceed human performance on tasks such as image classification (Fig. 4).

Nearly all scientific fields have felt the impact of this technology. Most industries and businesses have already been disrupted and transformed through the use of DL. The leading technology and economy-focused companies around the world are in a race to improve DL. Even now, human-level performance and capability cannot exceed that the performance of DL in many areas, such as predicting the time taken to make car deliveries, decisions to certify loan requests, and predicting movie ratings [38]. The winners of the 2019 “Nobel Prize” in computing, also known as the Turing Award, were three pioneers in the field of DL (Yann LeCun, Geoffrey Hinton, and Yoshua Bengio) [39]. Although a large number of goals have been achieved, there is further progress to be made in the DL context. In fact, DL has the ability to enhance human lives by providing additional accuracy in diagnosis, including estimating natural disasters [40], the discovery of new drugs [41], and cancer diagnosis [42–44]. Esteva et al. [45] found that a DL network has the same ability to diagnose the disease as twenty-one board-certified dermatologists using 129,450 images of 2032 diseases. Furthermore, in grading prostate cancer, US board-certified general pathologists achieved an average accuracy of 61%, while the Google AI [44] outperformed these specialists by achieving an average accuracy of 70%. In 2020, DL is playing an increasingly vital role in early diagnosis of the novel coronavirus (COVID-19) [29, 46–48]. DL has become the main tool in many hospitals around the world for automatic COVID-19 classification and detection using chest X-ray images or other types of images. We end this section by the saying of AI pioneer Geoffrey Hinton “Deep learning is going to be able to do everything”.

### When to apply deep learning

Machine intelligence is useful in many situations which is equal or better than human experts in some cases [49–52], meaning that DL can be a solution to the following problems:

- Cases where human experts are not available.
- Cases where humans are unable to explain decisions made using their expertise (language understanding, medical decisions, and speech recognition).
- Cases where the problem solution updates over time (price prediction, stock preference, weather prediction, and tracking).
- Cases where solutions require adaptation based on specific cases (personalization, biometrics).
- Cases where size of the problem is extremely large and exceeds our inadequate reasoning abilities (sentiment analysis, matching ads to Facebook, calculation webpage ranks).

### Why deep learning?

Several performance features may answer this question, e.g

1. Universal Learning Approach: Because DL has the ability to perform in approximately all application domains, it is sometimes referred to as universal learning.
2. Robustness: In general, precisely designed features are not required in DL techniques. Instead, the optimized features are learned in an automated fashion related to the task under consideration. Thus, robustness to the usual changes of the input data is attained.
3. Generalization: Different data types or different applications can use the same DL technique, an approach frequently referred to as transfer learning (TL) which explained in the latter section. Furthermore, it is a useful approach in problems where data is insufficient.
4. Scalability: DL is highly scalable. ResNet [37], which was invented by Microsoft, comprises 1202 layers and is frequently applied at a supercomputing scale. Lawrence Livermore National Laboratory (LLNL), a large enterprise working on evolving frameworks for networks, adopted a similar approach, where thousands of nodes can be implemented [53].

## Classification of DL approaches

DL techniques are classified into three major categories: unsupervised, partially supervised (semi-supervised) and supervised. Furthermore, deep reinforcement learning (DRL), also known as RL, is another type of learning technique, which is mostly considered to fall into the category of partially supervised (and occasionally unsupervised) learning techniques.

### Deep supervised learning

This technique deals with labeled data. When considering such a technique, the environs have a collection of inputs and resultant outputs $(x_t,y_t)\sim \rho$. For instance, the smart agent guesses  if the input is xt and will obtain  as a loss value. Next, the network parameters are repeatedly updated by the agent to obtain an improved estimate for the preferred outputs. Following a positive training outcome, the agent acquires the ability to obtain the right solutions to the queries from the environs. For DL, there are several supervised learning techniques, such as recurrent neural networks (RNNs), convolutional neural networks (CNNs), and deep neural networks (DNNs). In addition, the RNN category includes gated recurrent units (GRUs) and long short-term memory (LSTM) approaches. The main advantage of this technique is the ability to collect data or generate a data output from the prior knowledge. However, the disadvantage of this technique is that decision boundary might be overstrained when training set doesn’t own samples that should be in a class. Overall, this technique is simpler than other techniques in the way of learning with high performance.

### Deep semi-supervised learning

In this technique, the learning process is based on semi-labeled datasets. Occasionally, generative adversarial networks (GANs) and DRL are employed in the same way as this technique. In addition, RNNs, which include GRUs and LSTMs, are also employed for partially supervised learning. One of the advantages of this technique is to minimize the amount of labeled data needed. On other the hand, One of the disadvantages of this technique is irrelevant input feature present training data could furnish incorrect decisions. Text document classifier is one of the most popular example of an application of semi-supervised learning. Due to difficulty of obtaining a large amount of labeled text documents, semi-supervised learning is ideal for text document classification task.

### Deep unsupervised learning

This technique makes it possible to implement the learning process in the absence of available labeled data (i.e. no labels are required). Here, the agent learns the significant features or interior representation required to discover the unidentified structure or relationships in the input data. Techniques of generative networks, dimensionality reduction and clustering are frequently counted within the category of unsupervised learning. Several members of the DL family have performed well on non-linear dimensionality reduction and clustering tasks; these include restricted Boltzmann machines, auto-encoders and GANs as the most recently developed techniques. Moreover, RNNs, which include GRUs and LSTM approaches, have also been employed for unsupervised learning in a wide range of applications. The main disadvantages of unsupervised learning are unable to provide accurate information concerning data sorting and computationally complex. One of the most popular unsupervised learning approaches is clustering [54].

### Deep reinforcement learning

Reinforcement Learning operates on interacting with the environment, while supervised learning operates on provided sample data. This technique was developed in 2013 with Google Deep Mind [55]. Subsequently, many enhanced techniques dependent on reinforcement learning were constructed. For example, if the input environment samples: $x_t\sim \rho$, agent predict:  and the received cost of the agent is  , P here is the unknown probability distribution, then the environment asks a question to the agent. The answer it gives is a noisy score. This method is sometimes referred to as semi-supervised learning. Based on this concept, several supervised and unsupervised techniques were developed. In comparison with traditional supervised techniques, performing this learning is much more difficult, as no straightforward loss function is available in the reinforcement learning technique. In addition, there are two essential differences between supervised learning and reinforcement learning: first, there is no complete access to the function, which requires optimization, meaning that it should be queried via interaction; second, the state being interacted with is founded on an environment, where the input $x_t$ is based on the preceding actions [9, 56].

For solving a task, the selection of the type of reinforcement learning that needs to be performed is based on the space or the scope of the problem. For example, DRL is the best way for problems involving many parameters to be optimized. By contrast, derivative-free reinforcement learning is a technique that performs well for problems with limited parameters. Some of the applications of reinforcement learning are business strategy planning and robotics for industrial automation. The main drawback of Reinforcement Learning is that parameters may influence the speed of learning. Here are the main motivations for utilizing Reinforcement Learning:

- It assists you to identify which action produces the highest reward over a longer period.
- It assists you to discover which situation requires action.
- It also enables it to figure out the best approach for reaching large rewards.
- Reinforcement Learning also gives the learning agent a reward function.

Reinforcement Learning can’t utilize in all the situation such as:

- In case there is sufficient data to resolve the issue with supervised learning techniques.
- Reinforcement Learning is computing-heavy and time-consuming. Specially when the workspace is large.

## Types of DL networks

The most famous types of deep learning networks are discussed in this section: these include recursive neural networks (RvNNs), RNNs, and CNNs. RvNNs and RNNs were briefly explained in this section while CNNs were explained in deep due to the importance of this type. Furthermore, it is the most used in several applications among other networks.

### Recursive neural networks

**Fig. 5:**
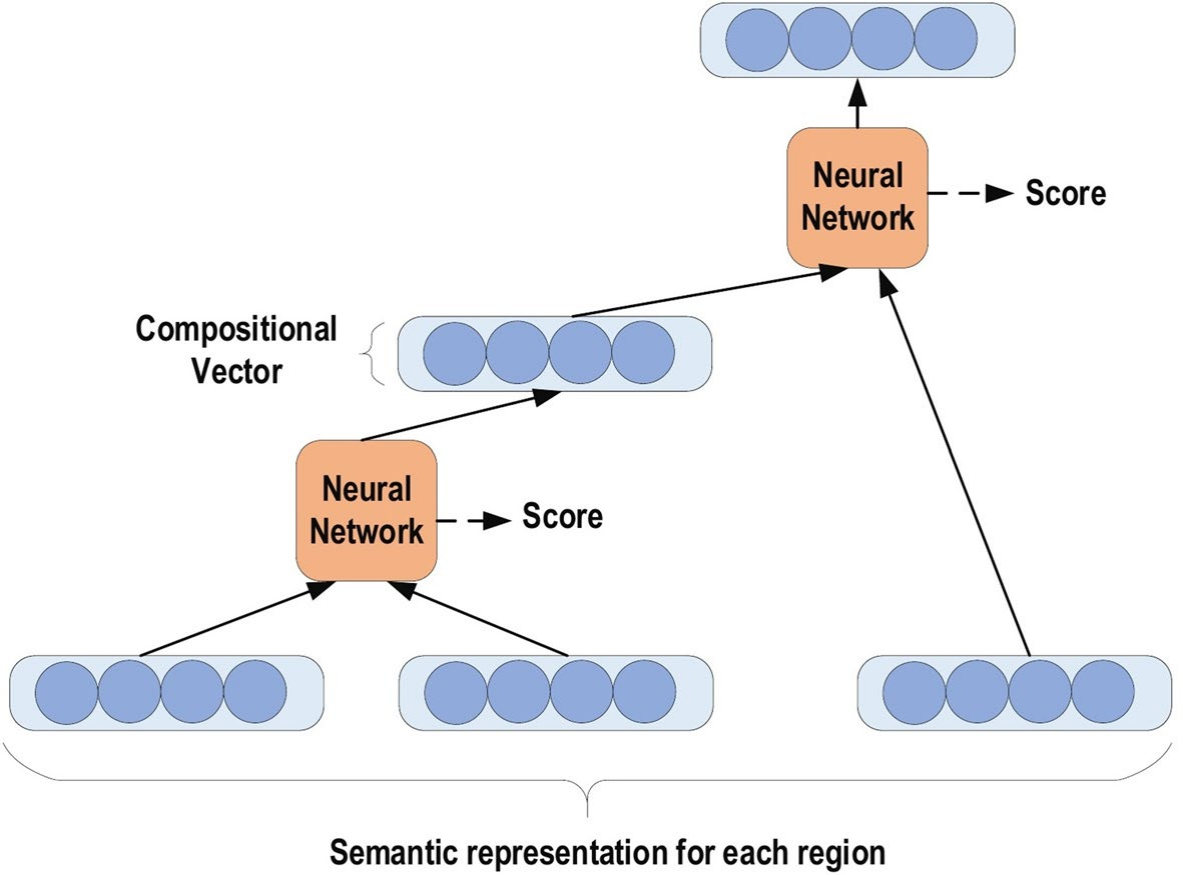
An example of RvNN tree

RvNN can achieve predictions in a hierarchical structure also classify the outputs utilizing compositional vectors [57]. Recursive auto-associative memory (RAAM) [58] is the primary inspiration for the RvNN development. The RvNN architecture is generated for processing objects, which have randomly shaped structures like graphs or trees. This approach generates a fixed-width distributed representation from a variable-size recursive-data structure. The network is trained using an introduced back-propagation through structure (BTS) learning system [58]. The BTS system tracks the same technique as the general-back propagation algorithm and has the ability to support a treelike structure. Auto-association trains the network to regenerate the input-layer pattern at the output layer. RvNN is highly effective in the NLP context. Socher et al. [59] introduced RvNN architecture designed to process inputs from a variety of modalities. These authors demonstrate two applications for classifying natural language sentences: cases where each sentence is split into words and nature images, and cases where each image is separated into various segments of interest. RvNN computes a likely pair of scores for merging and constructs a syntactic tree. Furthermore, RvNN calculates a score related to the merge plausibility for every pair of units. Next, the pair with the largest score is merged within a composition vector. Following every merge, RvNN generates (a) a larger area of numerous units, (b) a compositional vector of the area, and (c) a label for the class (for instance, a noun phrase will become the class label for the new area if two units are noun words). The compositional vector for the entire area is the root of the RvNN tree structure. An example RvNN tree is shown in Fig. 5. RvNN has been employed in several applications [60–62].

### Recurrent neural networks

**Fig. 6:**
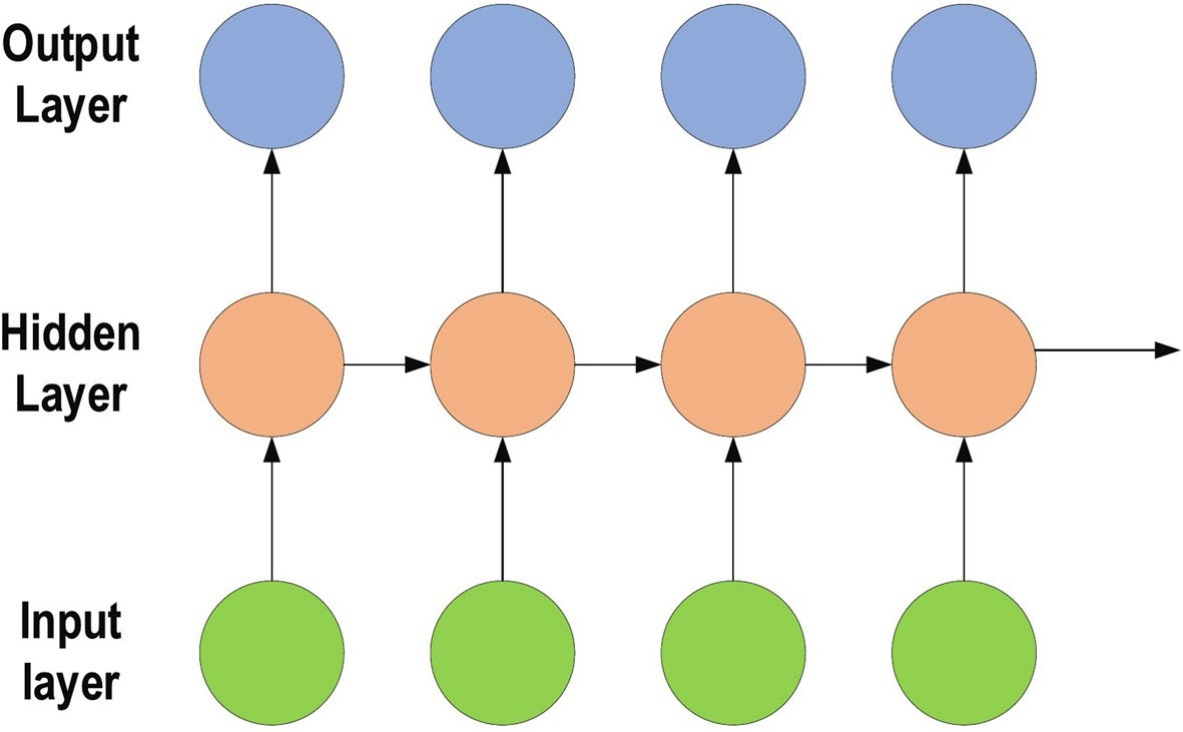
Typical unfolded RNN diagram

RNNs are a commonly employed and familiar algorithm in the discipline of DL [63–65]. RNN is mainly applied in the area of speech processing and NLP contexts [66, 67]. Unlike conventional networks, RNN uses sequential data in the network. Since the embedded structure in the sequence of the data delivers valuable information, this feature is fundamental to a range of different applications. For instance, it is important to understand the context of the sentence in order to determine the meaning of a specific word in it. Thus, it is possible to consider the RNN as a unit of short-term memory, where x represents the input layer, y is the output layer, and s represents the state (hidden) layer. For a given input sequence, a typical unfolded RNN diagram is illustrated in Fig. 6. Pascanu et al. [68] introduced three different types of deep RNN techniques, namely “Hidden-to-Hidden”, “Hidden-to-Output”, and “Input-to-Hidden”. A deep RNN is introduced that lessens the learning difficulty in the deep network and brings the benefits of a deeper RNN based on these three techniques.

However, RNN’s sensitivity to the exploding gradient and vanishing problems represent one of the main issues with this approach [69]. More specifically, during the training process, the reduplications of several large or small derivatives may cause the gradients to exponentially explode or decay. With the entrance of new inputs, the network stops thinking about the initial ones; therefore, this sensitivity decays over time. Furthermore, this issue can be handled using LSTM [70]. This approach offers recurrent connections to memory blocks in the network. Every memory block contains a number of memory cells, which have the ability to store the temporal states of the network. In addition, it contains gated units for controlling the flow of information. In very deep networks [37], residual connections also have the ability to considerably reduce the impact of the vanishing gradient issue which explained in later sections. CNN is considered to be more powerful than RNN. RNN includes less feature compatibility when compared to CNN.

### Convolutional neural networks

In the field of DL, the CNN is the most famous and commonly employed algorithm [30, 71–75]. The main benefit of CNN compared to its predecessors is that it automatically identifies the relevant features without any human supervision [76]. CNNs have been extensively applied in a range of different fields, including computer vision [77], speech processing [78], Face Recognition [79], etc. The structure of CNNs was inspired by neurons in human and animal brains, similar to a conventional neural network. More specifically, in a cat’s brain, a complex sequence of cells forms the visual cortex; this sequence is simulated by the CNN [80]. Goodfellow et al. [28] identified three key benefits of the CNN: equivalent representations, sparse interactions, and parameter sharing. Unlike conventional fully connected (FC) networks, shared weights and local connections in the CNN are employed to make full use of 2D input-data structures like image signals. This operation utilizes an extremely small number of parameters, which both simplifies the training process and speeds up the network. This is the same as in the visual cortex cells. Notably, only small regions of a scene are sensed by these cells rather than the whole scene (i.e., these cells spatially extract the local correlation available in the input, like local filters over the input).

**Fig. 7:**
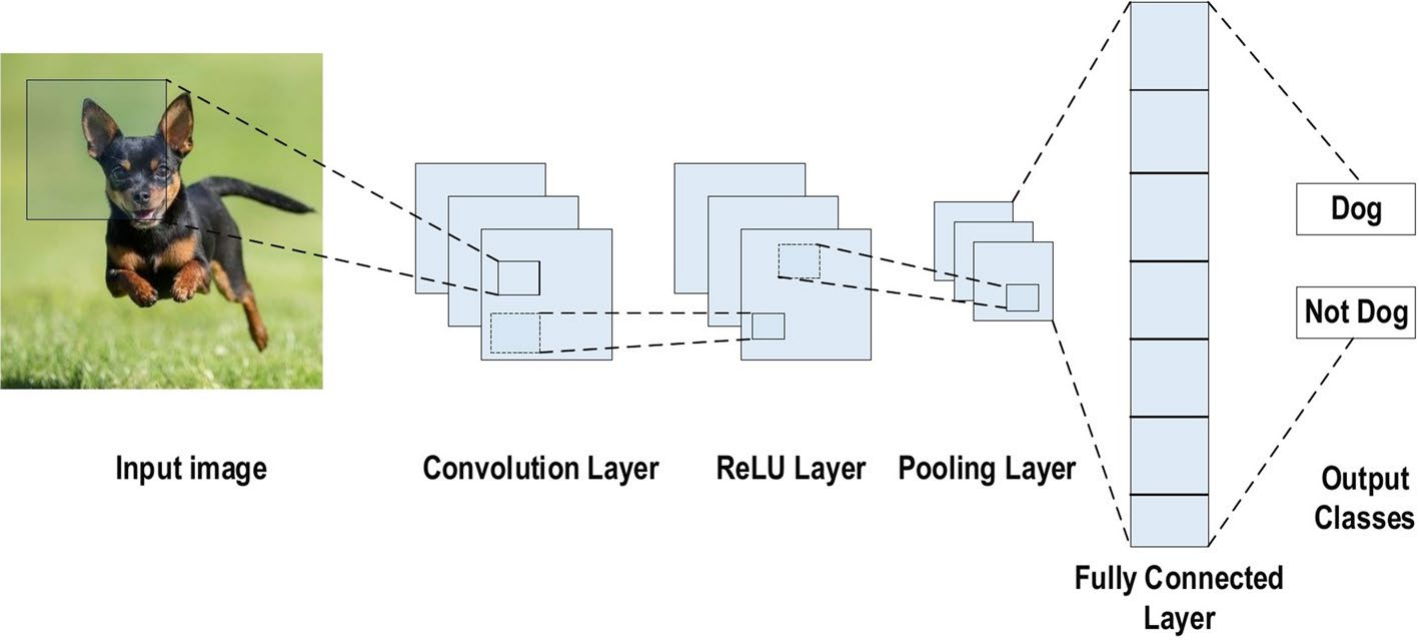
An example of CNN architecture for image classification

A commonly used type of CNN, which is similar to the multi-layer perceptron (MLP), consists of numerous convolution layers preceding sub-sampling (pooling) layers, while the ending layers are FC layers. An example of CNN architecture for image classification is illustrated in Fig. 7.

The input *x* of each layer in a CNN model is organized in three dimensions: height, width, and depth, or $m \times m \times r$, where the height (m) is equal to the width. The depth is also referred to as the channel number. For example, in an RGB image, the depth (r) is equal to three. Several kernels (filters) available in each convolutional layer are denoted by *k* and also have three dimensions ($n \times n \times q$), similar to the input image; here, however, *n* must be smaller than *m*, while *q* is either equal to or smaller than *r*. In addition, the kernels are the basis of the local connections, which share similar parameters (bias $b^{k}$ and weight $W^{k}$) for generating *k* feature maps $h^{k}$ with a size of ($m-n-1$) each and are convolved with input, as mentioned above. The convolution layer calculates a dot product between its input and the weights as in Eq. 1, similar to NLP, but the inputs are undersized areas of the initial image size. Next, by applying the nonlinearity or an activation function to the convolution-layer output, we obtain the following:

1 $$ h^{k}= f(W^{k}*x+ b^{k} )$$

The next step is down-sampling every feature map in the sub-sampling layers. This leads to a reduction in the network parameters, which accelerates the training process and in turn enables handling of the overfitting issue. For all feature maps, the pooling function (e.g. max or average) is applied to an adjacent area of size $p \times p$, where *p* is the kernel size. Finally, the FC layers receive the mid- and low-level features and create the high-level abstraction, which represents the last-stage layers as in a typical neural network. The classification scores are generated using the ending layer [e.g. support vector machines (SVMs) or softmax]. For a given instance, every score represents the probability of a specific class.

#### Benefits of employing CNNs

The benefits of using CNNs over other traditional neural networks in the computer vision environment are listed as follows:

1. The main reason to consider CNN is the weight sharing feature, which reduces the number of trainable network parameters and in turn helps the network to enhance generalization and to avoid overfitting.
2. Concurrently learning the feature extraction layers and the classification layer causes the model output to be both highly organized and highly reliant on the extracted features.
3. Large-scale network implementation is much easier with CNN than with other neural networks.

#### CNN layers

The CNN architecture consists of a number of layers (or so-called multi-building blocks). Each layer in the CNN architecture, including its function, is described in detail below.

1. Convolutional Layer: In CNN architecture, the most significant component is the convolutional layer. It consists of a collection of convolutional filters (so-called kernels). The input image, expressed as N-dimensional metrics, is convolved with these filters to generate the output feature map.
  Kernel definition: A grid of discrete numbers or values describes the kernel. Each value is called the kernel weight. Random numbers are assigned to act as the weights of the kernel at the beginning of the CNN training process. In addition, there are several different methods used to initialize the weights. Next, these weights are adjusted at each training era; thus, the kernel learns to extract significant features.
  Convolutional Operation: Initially, the CNN input format is described. The vector format is the input of the traditional neural network, while the multi-channeled image is the input of the CNN. For instance, single-channel is the format of the gray-scale image, while the RGB image format is three-channeled. To understand the convolutional operation, let us take an example of a $4 \times 4$ gray-scale image with a $2 \times 2$ random weight-initialized kernel. First, the kernel slides over the whole image horizontally and vertically. In addition, the dot product between the input image and the kernel is determined, where their corresponding values are multiplied and then summed up to create a single scalar value, calculated concurrently. The whole process is then repeated until no further sliding is possible. Note that the calculated dot product values represent the feature map of the output. Figure 8 graphically illustrates the primary calculations executed at each step. In this figure, the light green color represents the $2 \times 2$ kernel, while the light blue color represents the similar size area of the input image. Both are multiplied; the end result after summing up the resulting product values (marked in a light orange color) represents an entry value to the output feature map.

**Fig. 8:**
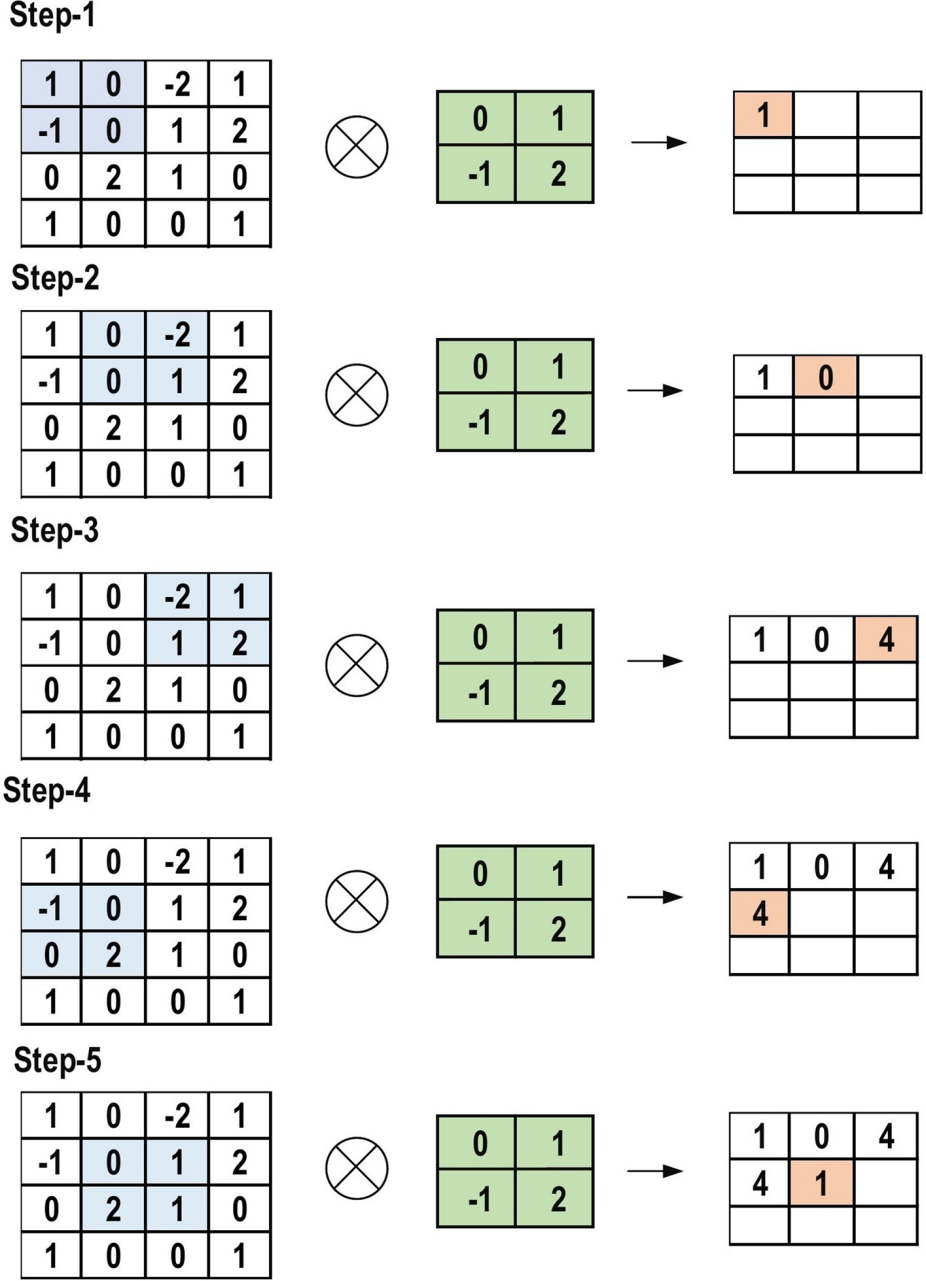
The primary calculations executed at each step of convolutional layer
    However, padding to the input image is not applied in the previous example, while a stride of one (denoted for the selected step-size over all vertical or horizontal locations) is applied to the kernel. Note that it is also possible to use another stride value. In addition, a feature map of lower dimensions is obtained as a result of increasing the stride value.
    On the other hand, padding is highly significant to determining border size information related to the input image. By contrast, the border side-features moves carried away very fast. By applying padding, the size of the input image will increase, and in turn, the size of the output feature map will also increase. Core Benefits of Convolutional Layers.
    Sparse Connectivity: Each neuron of a layer in FC neural networks links with all neurons in the following layer. By contrast, in CNNs, only a few weights are available between two adjacent layers. Thus, the number of required weights or connections is small, while the memory required to store these weights is also small; hence, this approach is memory-effective. In addition, matrix operation is computationally much more costly than the dot (.) operation in CNN.
    Weight Sharing: There are no allocated weights between any two neurons of neighboring layers in CNN, as the whole weights operate with one and all pixels of the input matrix. Learning a single group of weights for the whole input will significantly decrease the required training time and various costs, as it is not necessary to learn additional weights for each neuron.
2. Pooling Layer: The main task of the pooling layer is the sub-sampling of the feature maps. These maps are generated by following the convolutional operations. In other words, this approach shrinks large-size feature maps to create smaller feature maps. Concurrently, it maintains the majority of the dominant information (or features) in every step of the pooling stage. In a similar manner to the convolutional operation, both the stride and the kernel are initially size-assigned before the pooling operation is executed. Several types of pooling methods are available for utilization in various pooling layers. These methods include tree pooling, gated pooling, average pooling, min pooling, max pooling, global average pooling (GAP), and global max pooling. The most familiar and frequently utilized pooling methods are the max, min, and GAP pooling. Figure 9 illustrates these three pooling operations.

**Fig. 9:**
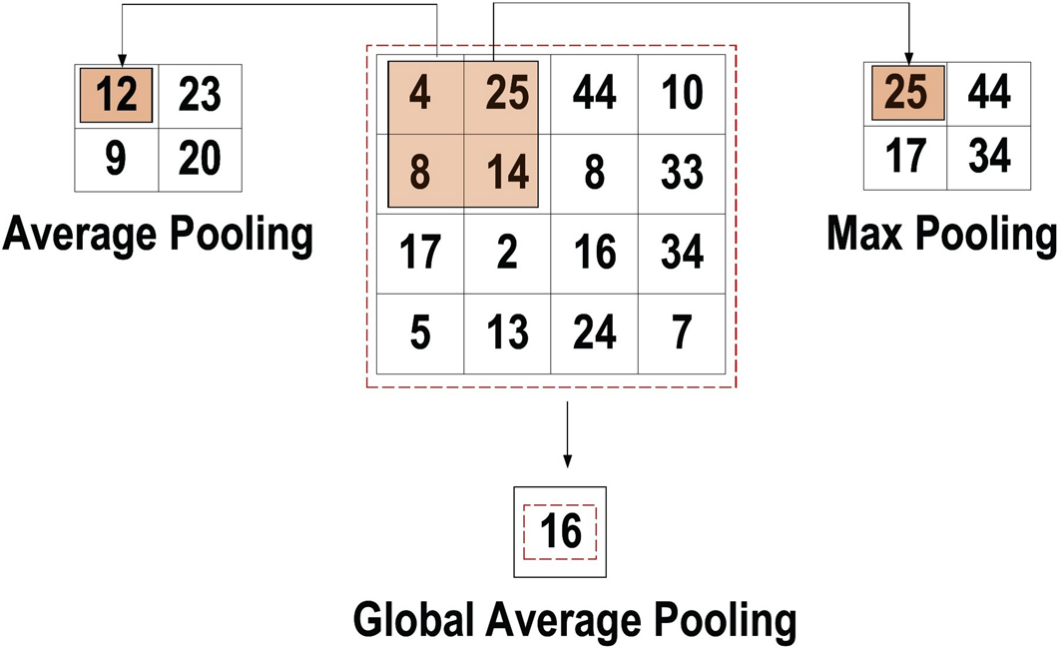
Three types of pooling operations
  Sometimes, the overall CNN performance is decreased as a result; this represents the main shortfall of the pooling layer, as this layer helps the CNN to determine whether or not a certain feature is available in the particular input image, but focuses exclusively on ascertaining the correct location of that feature. Thus, the CNN model misses the relevant information.
3. Activation Function (non-linearity) Mapping the input to the output is the core function of all types of activation function in all types of neural network. The input value is determined by computing the weighted summation of the neuron input along with its bias (if present). This means that the activation function makes the decision as to whether or not to fire a neuron with reference to a particular input by creating the corresponding output.
  Non-linear activation layers are employed after all layers with weights (so-called learnable layers, such as FC layers and convolutional layers) in CNN architecture. This non-linear performance of the activation layers means that the mapping of input to output will be non-linear; moreover, these layers give the CNN the ability to learn extra-complicated things. The activation function must also have the ability to differentiate, which is an extremely significant feature, as it allows error back-propagation to be used to train the network. The following types of activation functions are most commonly used in CNN and other deep neural networks.
  Sigmoid: The input of this activation function is real numbers, while the output is restricted to between zero and one. The sigmoid function curve is S-shaped and can be represented mathematically by Eq. 2.
    2 $$ f(x)_{sigm}=\frac{1}{1+e^{-x}} $$
  Tanh: It is similar to the sigmoid function, as its input is real numbers, but the output is restricted to between − 1 and 1. Its mathematical representation is in Eq. 3.
    3 $$ f(x)_{tanh}=\frac{e^{x}-e^{-x}}{e^{x}+e^{-x}} $$
  ReLU: The mostly commonly used function in the CNN context. It converts the whole values of the input to positive numbers. Lower computational load is the main benefit of ReLU over the others. Its mathematical representation is in Eq. 4.
    4 $$ f(x)_{ReLU}= max(0,x) $$
    Occasionally, a few significant issues may occur during the use of ReLU. For instance, consider an error back-propagation algorithm with a larger gradient flowing through it. Passing this gradient within the ReLU function will update the weights in a way that makes the neuron certainly not activated once more. This issue is referred to as “Dying ReLU”. Some ReLU alternatives exist to solve such issues. The following discusses some of them.
  Leaky ReLU: Instead of ReLU down-scaling the negative inputs, this activation function ensures these inputs are never ignored. It is employed to solve the Dying ReLU problem. Leaky ReLU can be represented mathematically as in Eq. 5.
    5 $$\begin{aligned} f(x)_{Leaky ReLU}= \left \{ \begin{array}{ll} x,& if \quad  x > 0\\ mx,& x \le 0 \end{array} \right \} \end{aligned}$$
    Note that the leak factor is denoted by m. It is commonly set to a very small value, such as 0.001.
  Noisy ReLU: This function employs a Gaussian distribution to make ReLU noisy. It can be represented mathematically as in Eq. 6.
    6 $$ f(x)_{Noisy ReLU}= max(x+Y),with\, Y \sim N (0,\sigma (x)) $$
  Parametric Linear Units: This is mostly the same as Leaky ReLU. The main difference is that the leak factor in this function is updated through the model training process. The parametric linear unit can be represented mathematically as in Eq. 7.
    7 $$\begin{aligned} f(x)_{ Parametric Linear}=\begin{Bmatrix} x,& if\; x >0\\ ax,& x \le 0 \end{Bmatrix} \end{aligned}$$
    Note that the learnable weight is denoted as a.
4. Fully Connected Layer: Commonly, this layer is located at the end of each CNN architecture. Inside this layer, each neuron is connected to all neurons of the previous layer, the so-called Fully Connected (FC) approach. It is utilized as the CNN classifier. It follows the basic method of the conventional multiple-layer perceptron neural network, as it is a type of feed-forward ANN. The input of the FC layer comes from the last pooling or convolutional layer. This input is in the form of a vector, which is created from the feature maps after flattening. The output of the FC layer represents the final CNN output, as illustrated in Fig. 10.

**Fig. 10:**
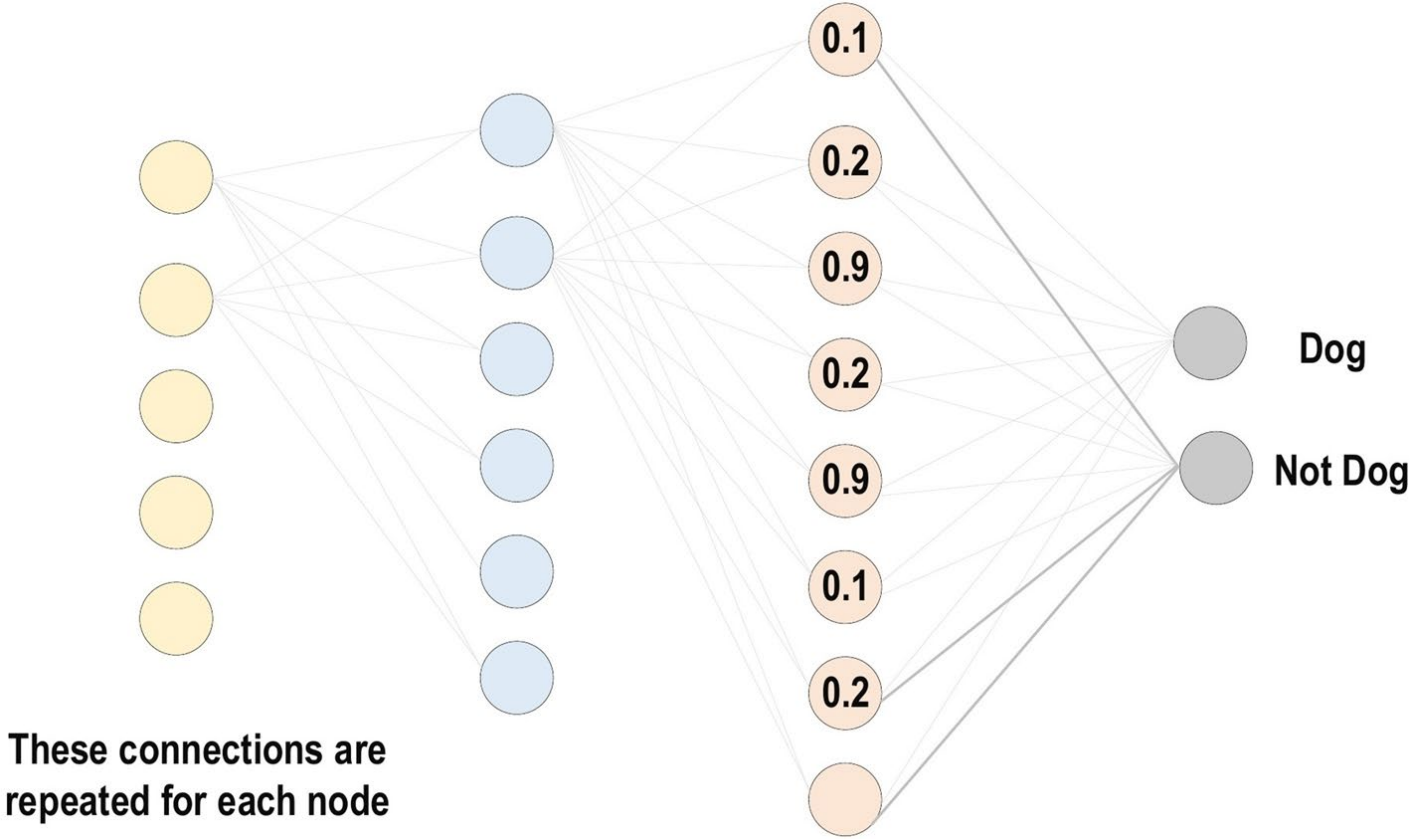
Fully connected layer
5. Loss Functions: The previous section has presented various layer-types of CNN architecture. In addition, the final classification is achieved from the output layer, which represents the last layer of the CNN architecture. Some loss functions are utilized in the output layer to calculate the predicted error created across the training samples in the CNN model. This error reveals the difference between the actual output and the predicted one. Next, it will be optimized through the CNN learning process.
  However, two parameters are used by the loss function to calculate the error. The CNN estimated output (referred to as the prediction) is the first parameter. The actual output (referred to as the label) is the second parameter. Several types of loss function are employed in various problem types. The following concisely explains some of the loss function types.
  - Cross-Entropy or Softmax Loss Function: This function is commonly employed for measuring the CNN model performance. It is also referred to as the log loss function. Its output is the probability $p \in \left\{ 0\left. , 1 \right\} \right.$. In addition, it is usually employed as a substitution of the square error loss function in multi-class classification problems. In the output layer, it employs the softmax activations to generate the output within a probability distribution. The mathematical representation of the output class probability is Eq. 8.
    8 $$ p_{i}= \frac{e^{a_{i}}}{\sum _{k=1}^{N} e^{a}_{_{k}}}$$
    Here, $e^{a_{i}}$ represents the non-normalized output from the preceding layer, while *N* represents the number of neurons in the output layer. Finally, the mathematical representation of cross-entropy loss function is Eq. 9.
    9 $$ H(p,y)=-\sum _{i}^{} y_{i}\log (p_{i}) \quad where \quad i \in [1,N] $$
  - Euclidean Loss Function: This function is widely used in regression problems. In addition, it is also the so-called mean square error. The mathematical expression of the estimated Euclidean loss is Eq. 10.
    10 $$ H(p,y)=\frac{1}{2N}\sum _{i=1}^{N} (p_{i}-y_{i})^{2} $$
  - Hinge Loss Function: This function is commonly employed in problems related to binary classification. This problem relates to maximum-margin-based classification; this is mostly important for SVMs, which use the hinge loss function, wherein the optimizer attempts to maximize the margin around dual objective classes. Its mathematical formula is Eq. 11.
    11 $$ H(p,y)=\sum _{i=1}^{N} max (0,m-(2y_{i}-1)p_{_{i}}) $$
    The margin *m* is commonly set to 1. Moreover, the predicted output is denoted as $p_{_{i}}$, while the desired output is denoted as $y_{_{i}}$.

#### Regularization to CNN

**Fig. 11:**
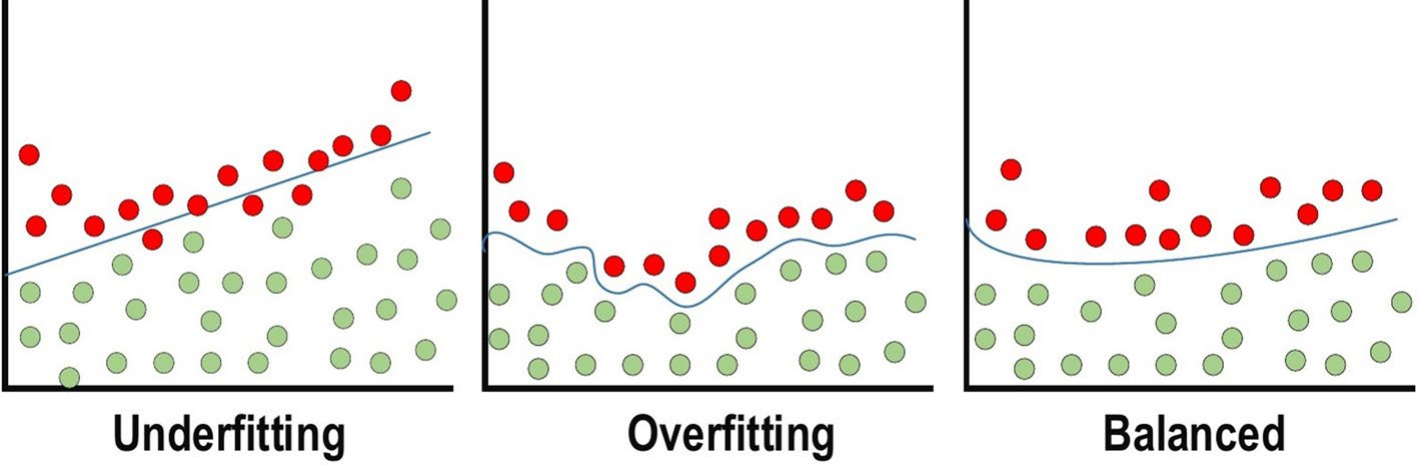
Over-fitting and under-fitting issues

For CNN models, over-fitting represents the central issue associated with obtaining well-behaved generalization. The model is entitled over-fitted in cases where the model executes especially well on training data and does not succeed on test data (unseen data) which is more explained in the latter section. An under-fitted model is the opposite; this case occurs when the model does not learn a sufficient amount from the training data. The model is referred to as “just-fitted” if it executes well on both training and testing data. These three types are illustrated in Fig. 11. Various intuitive concepts are used to help the regularization to avoid over-fitting; more details about over-fitting and under-fitting are discussed in latter sections.

1. Dropout: This is a widely utilized technique for generalization. During each training epoch, neurons are randomly dropped. In doing this, the feature selection power is distributed equally across the whole group of neurons, as well as forcing the model to learn different independent features. During the training process, the dropped neuron will not be a part of back-propagation or forward-propagation. By contrast, the full-scale network is utilized to perform prediction during the testing process.
2. Drop-Weights: This method is highly similar to dropout. In each training epoch, the connections between neurons (weights) are dropped rather than dropping the neurons; this represents the only difference between drop-weights and dropout.
3. Data Augmentation: Training the model on a sizeable amount of data is the easiest way to avoid over-fitting. To achieve this, data augmentation is used. Several techniques are utilized to artificially expand the size of the training dataset. More details can be found in the latter section, which describes the data augmentation techniques.
4. Batch Normalization: This method ensures the performance of the output activations [81]. This performance follows a unit Gaussian distribution. Subtracting the mean and dividing by the standard deviation will normalize the output at each layer. While it is possible to consider this as a pre-processing task at each layer in the network, it is also possible to differentiate and to integrate it with other networks. In addition, it is employed to reduce the “internal covariance shift” of the activation layers. In each layer, the variation in the activation distribution defines the internal covariance shift. This shift becomes very high due to the continuous weight updating through training, which may occur if the samples of the training data are gathered from numerous dissimilar sources (for example, day and night images). Thus, the model will consume extra time for convergence, and in turn, the time required for training will also increase. To resolve this issue, a layer representing the operation of batch normalization is applied in the CNN architecture.
  The advantages of utilizing batch normalization are as follows:
  It prevents the problem of vanishing gradient from arising.
  It can effectively control the poor weight initialization.
  It significantly reduces the time required for network convergence (for large-scale datasets, this will be extremely useful).
  It struggles to decrease training dependency across hyper-parameters.
  Chances of over-fitting are reduced, since it has a minor influence on regularization.

#### Optimizer selection

This section discusses the CNN learning process. Two major issues are included in the learning process: the first issue is the learning algorithm selection (optimizer), while the second issue is the use of many enhancements (such as AdaDelta, Adagrad, and momentum) along with the learning algorithm to enhance the output.

Loss functions, which are founded on numerous learnable parameters (e.g. biases, weights, etc.) or minimizing the error (variation between actual and predicted output), are the core purpose of all supervised learning algorithms. The techniques of gradient-based learning for a CNN network appear as the usual selection. The network parameters should always update though all training epochs, while the network should also look for the locally optimized answer in all training epochs in order to minimize the error.

The learning rate is defined as the step size of the parameter updating. The training epoch represents a complete repetition of the parameter update that involves the complete training dataset at one time. Note that it needs to select the learning rate wisely so that it does not influence the learning process imperfectly, although it is a hyper-parameter.

Gradient Descent or Gradient-based learning algorithm: To minimize the training error, this algorithm repetitively updates the network parameters through every training epoch. More specifically, to update the parameters correctly, it needs to compute the objective function gradient (slope) by applying a first-order derivative with respect to the network parameters. Next, the parameter is updated in the reverse direction of the gradient to reduce the error. The parameter updating process is performed though network back-propagation, in which the gradient at every neuron is back-propagated to all neurons in the preceding layer. The mathematical representation of this operation is as Eq. 12.

12 $$ w_{i j^{t}}=w_{i j^{t-1}}-\Delta w_{i j^{t}},\quad \Delta w_{i j^{t}}=\eta *\frac{\partial E}{\partial w_{i j}} $$

The final weight in the current training epoch is denoted by $w_{i j^{t}}$, while the weight in the preceding $(t-1)$ training epoch is denoted $w_{i j^{t-1}}$. The learning rate is $\eta$ and the prediction error is *E*. Different alternatives of the gradient-based learning algorithm are available and commonly employed; these include the following:

1. Batch Gradient Descent: During the execution of this technique [82], the network parameters are updated merely one time behind considering all training datasets via the network. In more depth, it calculates the gradient of the whole training set and subsequently uses this gradient to update the parameters. For a small-sized dataset, the CNN model converges faster and creates an extra-stable gradient using BGD. Since the parameters are changed only once for every training epoch, it requires a substantial amount of resources. By contrast, for a large training dataset, additional time is required for converging, and it could converge to a local optimum (for non-convex instances).
2. Stochastic Gradient Descent: The parameters are updated at each training sample in this technique [83]. It is preferred to arbitrarily sample the training samples in every epoch in advance of training. For a large-sized training dataset, this technique is both more memory-effective and much faster than BGD. However, because it is frequently updated, it takes extremely noisy steps in the direction of the answer, which in turn causes the convergence behavior to become highly unstable.
3. Mini-batch Gradient Descent: In this approach, the training samples are partitioned into several mini-batches, in which every mini-batch can be considered an under-sized collection of samples with no overlap between them [84]. Next, parameter updating is performed following gradient computation on every mini-batch. The advantage of this method comes from combining the advantages of both BGD and SGD techniques. Thus, it has a steady convergence, more computational efficiency and extra memory effectiveness. The following describes several enhancement techniques in gradient-based learning algorithms (usually in SGD), which further powerfully enhance the CNN training process.
4. Momentum: For neural networks, this technique is employed in the objective function. It enhances both the accuracy and the training speed by summing the computed gradient at the preceding training step, which is weighted via a factor $\lambda$ (known as the momentum factor). However, it therefore simply becomes stuck in a local minimum rather than a global minimum. This represents the main disadvantage of gradient-based learning algorithms. Issues of this kind frequently occur if the issue has no convex surface (or solution space).
  Together with the learning algorithm, momentum is used to solve this issue, which can be expressed mathematically as in Eq. 13.
  13 $$ \Delta w_{i j^{t}}= \left( \eta *\frac{\partial E}{\partial w_{i j}}\right) +(\lambda *\Delta w_{i j^{t-1}}) $$
  The weight increment in the current $t^{\prime} \text{th}$ training epoch is denoted as $\Delta w_{i j^{t}}$, while $\eta$ is the learning rate, and the weight increment in the preceding $(t-1)^{\prime} \text{th}$ training epoch. The momentum factor value is maintained within the range 0 to 1; in turn, the step size of the weight updating increases in the direction of the bare minimum to minimize the error. As the value of the momentum factor becomes very low, the model loses its ability to avoid the local bare minimum. By contrast, as the momentum factor value becomes high, the model develops the ability to converge much more rapidly. If a high value of momentum factor is used together with LR, then the model could miss the global bare minimum by crossing over it.
  However, when the gradient varies its direction continually throughout the training process, then the suitable value of the momentum factor (which is a hyper-parameter) causes a smoothening of the weight updating variations.
5. Adaptive Moment Estimation (Adam): It is another optimization technique or learning algorithm that is widely used. Adam [85] represents the latest trends in deep learning optimization. This is represented by the Hessian matrix, which employs a second-order derivative. Adam is a learning strategy that has been designed specifically for training deep neural networks. More memory efficient and less computational power are two advantages of Adam. The mechanism of Adam is to calculate adaptive LR for each parameter in the model. It integrates the pros of both Momentum and RMSprop. It utilizes the squared gradients to scale the learning rate as RMSprop and it is similar to the momentum by using the moving average of the gradient. The equation of Adam is represented in Eq. 14.
  14 $$ w_{i j^{t}}=w_{i j^{t-1}}-\frac{\eta }{\sqrt{\widehat{E[\delta ^{2}]^{t}} }+\in } *\widehat{E[\delta ^{2}]^{t}} $$

#### Design of algorithms (backpropagation)

**Fig. 12:**
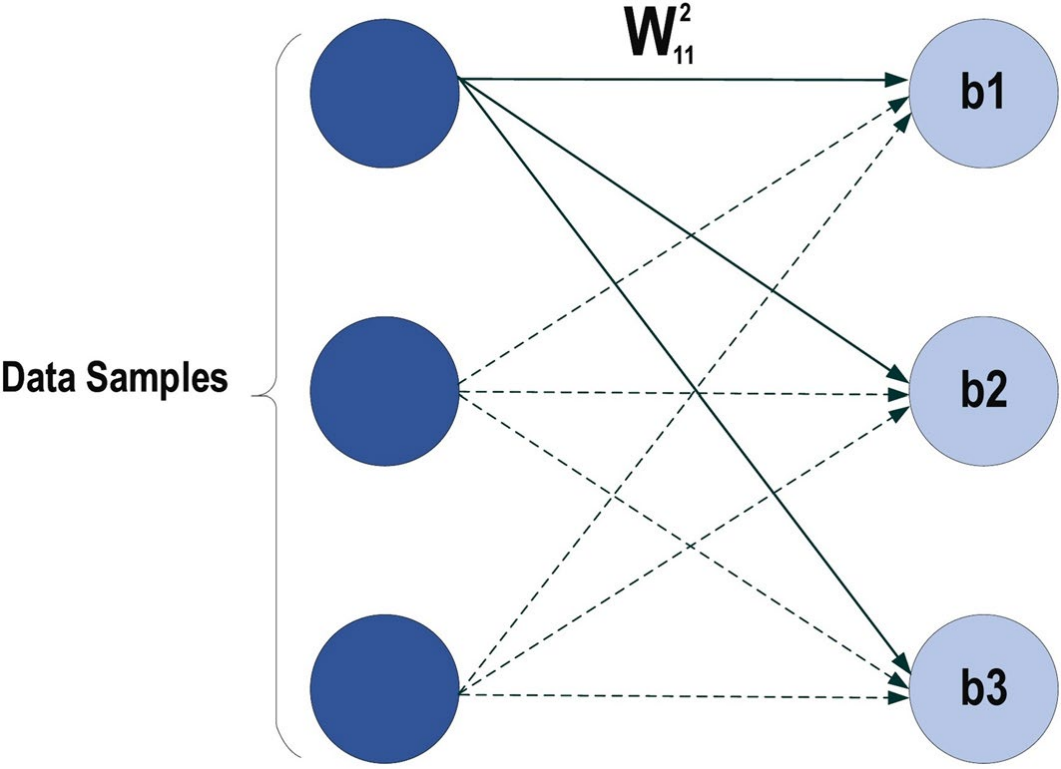
MLP structure

Let’s start with a notation that refers to weights in the network unambiguously. We denote ${\varvec{w}}_{i j}^{h}$ to be the weight for the connection from $\text {ith}$ input or (neuron at $\left. (\text {h}-1){\text{th}}\right)$ to the $j{\text{t }}$ neuron in the $\text {hth}$ layer. So, Fig. 12 shows the weight on a connection from the neuron in the first layer to another neuron in the next layer in the network.

**Fig. 13:**
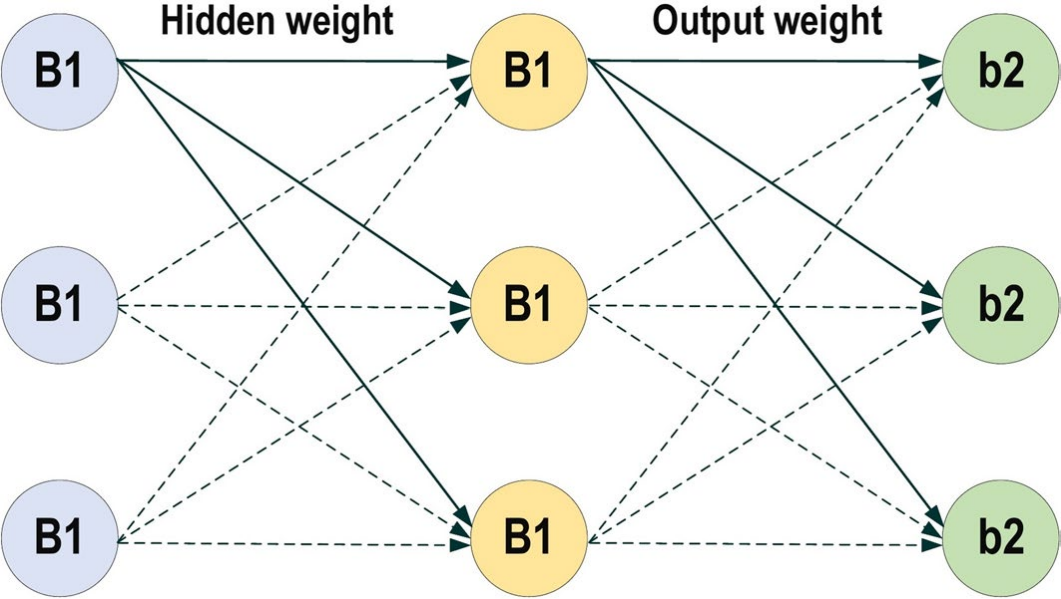
Neuron activation functions

Where $w_{11}^{2}$ has represented the weight from the first neuron in the first layer to the first neuron in the second layer, based on that the second weight for the same neuron will be $w_{21}^{2}$ which means is the weight comes from the second neuron in the previous layer to the first layer in the next layer which is the second in this net. Regarding the bias, since the bias is not the connection between the neurons for the layers, so it is easily handled each neuron must have its own bias, some network each layer has a certain bias. It can be seen from the above net that each layer has its own bias. Each network has the parameters such as the no of the layer in the net, the number of the neurons in each layer, no of the weight (connection) between the layers, the no of connection can be easily determined based on the no of neurons in each layer, for example, if there are ten input fully connect with two neurons in the next layer then the number of connection between them is $(10 * 2=20$ connection, weights), how the error is defined, and the weight is updated, we will imagine there is there are two layers in our neural network,

15 $$ \text{ error } =1 / 2\left( {\mathbf {d}}_{i}-{\mathbf {y}}_{i}\right) ^{2} $$

where $\text {d}$ is the label of induvial input $\text {ith}$ and $\text {y}$ is the output of the same individual input. Backpropagation is about understanding how to change the weights and biases in a network based on the changes of the cost function (Error). Ultimately, this means computing the partial derivatives $\partial \text {E} / \partial \text {w}_{\text {ij}}^{h}$ and $\partial \text {E} / \partial \text {b}_{\text {j}}^{h}.$ But to compute those, a local variable is introduced, $\delta _{j}^{1}$ which is called the local error in the $j{\text{th} }$ neuron in the $h{\text{th} }$ layer. Based on that local error Backpropagation will give the procedure to compute $\partial \text {E} / \partial \text {w}_{\text {ij}}^{h}$ and $\partial \text {E} / \partial \text {b}_{\text {j}}^{h}$ how the error is defined, and the weight is updated, we will imagine there is there are two layers in our neural network that is shown in Fig. 13.

Output error for $\delta _{\text {j}}^{1}$ each $1=1: \text {L}$ where $\text {L}$ is no. of neuron in output

16 $$ \delta _{\text {j}}^{1}({\mathbf {k}})=(-1) \text {e}(\text {k}) \varvec{\vartheta }^{\prime }\left( v_{j}({\varvec{k}})\right) $$

where $\text {e}(\text {k})$ is the error of the epoch $\text {k}$ as shown in Eq. (2) and $\varvec{\vartheta }^{\prime }\left( {\varvec{v}}_{j}({\varvec{k}})\right)$ is the derivate of the activation function for $v_{j}$ at the output.

Backpropagate the error at all the rest layer except the output

17 $$ \delta _{\text {j}}^{\text {h}}({\mathbf {k}}) =\varvec{\vartheta }^{\prime }\left( v_{j}({\varvec{k}})\right) \sum _{l=1}^{L} \delta _{\text {j}}^{1} \quad {\varvec{w}}_{j l}^{h+1}({\varvec{k}})$$

where $\delta _{j}^{1}({\mathbf {k}})$ is the output error and $w_{j l}^{h+1}(k)$ is represented the weight after the layer where the error need to obtain.

After finding the error at each neuron in each layer, now we can update the weight in each layer based on Eqs. (16) and (17).

#### Improving performance of CNN

Based on our experiments in different DL applications [86–88]. We can conclude the most active solutions that may improve the performance of CNN are:

- Expand the dataset with data augmentation or use transfer learning (explained in latter sections).
- Increase the training time.
- Increase the depth (or width) of the model.
- Add regularization.
- Increase hyperparameters tuning.

## CNN architectures

Over the last 10 years, several CNN architectures have been presented [21, 26]. Model architecture is a critical factor in improving the performance of different applications. Various modifications have been achieved in CNN architecture from 1989 until today. Such modifications include structural reformulation, regularization, parameter optimizations, etc. Conversely, it should be noted that the key upgrade in CNN performance occurred largely due to the processing-unit reorganization, as well as the development of novel blocks. In particular, the most novel developments in CNN architectures were performed on the use of network depth. In this section, we review the most popular CNN architectures, beginning from the AlexNet model in 2012 and ending at the High-Resolution (HR) model in 2020. Studying these architectures features (such as input size, depth, and robustness) is the key to help researchers to choose the suitable architecture for the their target task. Table 2 presents the brief overview of CNN architectures.

**Table 2:** Brief overview of CNN architectures

Model	Main finding	Depth	Dataset	Error rate	Input size	Year
AlexNet	Utilizes Dropout and ReLU	8	ImageNet	16.4	$227 \times 227 \times 3$	2012
NIN	New layer, called ‘mlpconv’, utilizes GAP	3	CIFAR-10, CIFAR-100, MNIST	10.41, 35.68, 0.45	$32 \times 32 \times 3$	2013
ZfNet	Visualization idea of middle layers	8	ImageNet	11.7	$224 \times 224 \times 3$	2014
VGG	Increased depth, small filter size	16, 19	ImageNet	7.3	$224 \times 224 \times 3$	2014
GoogLeNet	Increased depth,block concept, different filter size, concatenation concept	22	ImageNet	6.7	$224 \times 224 \times 3$	2015
Inception-V3	Utilizes small filtersize, better feature representation	48	ImageNet	3.5	$229 \times 229 \times 3$	2015
Highway	Presented the multipath concept	19, 32	CIFAR-10	7.76	$32 \times 32 \times 3$	2015
Inception-V4	Divided transform and integration concepts	70	ImageNet	3.08	$229 \times 229 \times 3$	2016
ResNet	Robust against overfitting due to symmetry mapping-based skip links	152	ImageNet	3.57	$224 \times 224 \times 3$	2016
Inception-ResNet-v2	Introduced the concept of residual links	164	ImageNet	3.52	$229 \times 229 \times 3$	2016
FractalNet	Introduced the concept of Drop-Path as regularization	40,80	CIFAR-10	4.60	$32 \times 32 \times 3$	2016
			CIFAR-100	18.85		
WideResNet	Decreased the depth and increased the width	28	CIFAR-10	3.89	$32 \times 32 \times 3$	2016
			CIFAR-100	18.85		
Xception	A depthwise convolutionfollowed by a pointwise convolution	71	ImageNet	0.055	$229 \times 229 \times 3$	2017
Residual attention neural network	Presented the attention technique	452	CIFAR-10, CIFAR-100	3.90, 20.4	$40 \times 40\times 3$	2017
Squeeze-and-excitation networks	Modeled interdependencies between channels	152	ImageNet	2.25	$229 \times 229 \times 3$	2017
					$$224 \times 224 \times 3$$	
					$$320 \times 320 \times 3$$	
DenseNet	Blocks of layers; layers connected to each other	201	CIFAR-10, CIFAR-100,ImageNet	3.46, 17.18, 5.54	$224 \times 224 \times 3$	2017
Competitive squeeze and excitation network	Both residual and identity mappings utilized to rescale the channel	152	CIFAR-10	3.58	$32 \times 32 \times 3$	2018
			CIFAR-100	18.47		
MobileNet-v2	Inverted residual structure	53	ImageNet	–	$224 \times 224 \times 3$	2018
CapsuleNet	Pays attention to special relationships between features	3	MNIST	0.00855	$28 \times 28 \times 1$	2018
HRNetV2	High-resolution representations	–	ImageNet	5.4	$224 \times 224 \times 3$	2020

### AlexNet

**Fig. 14:**
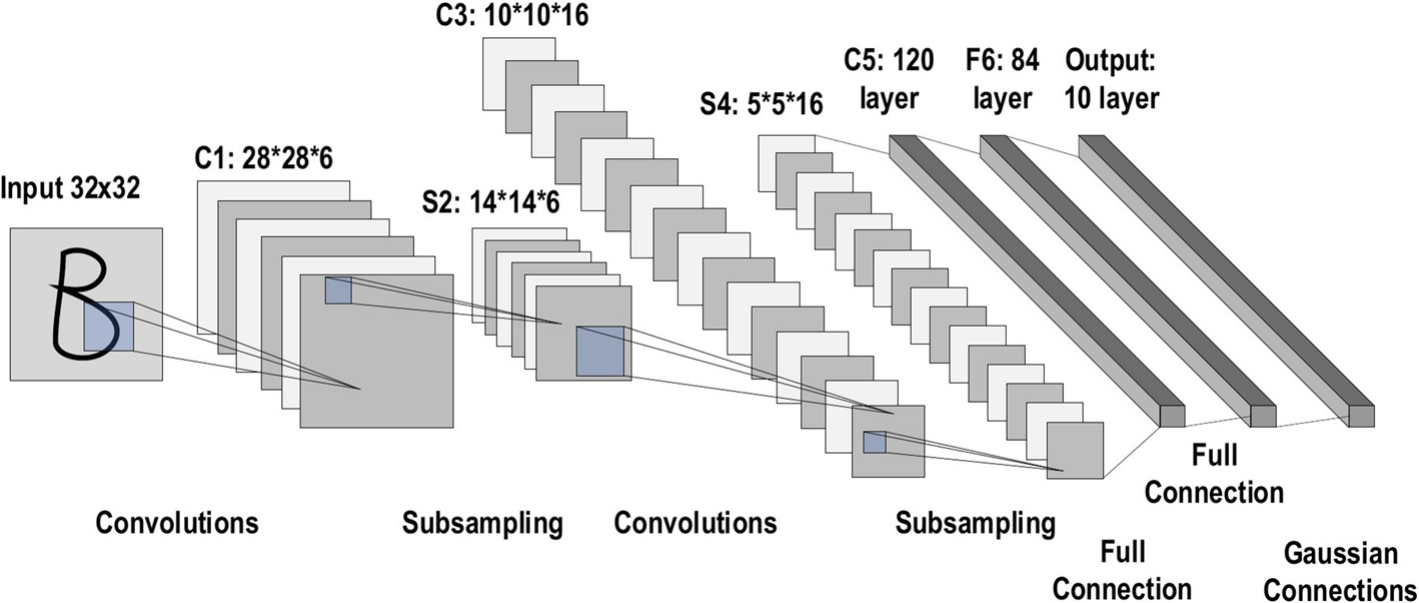
The architecture of LeNet

**Fig. 15:**
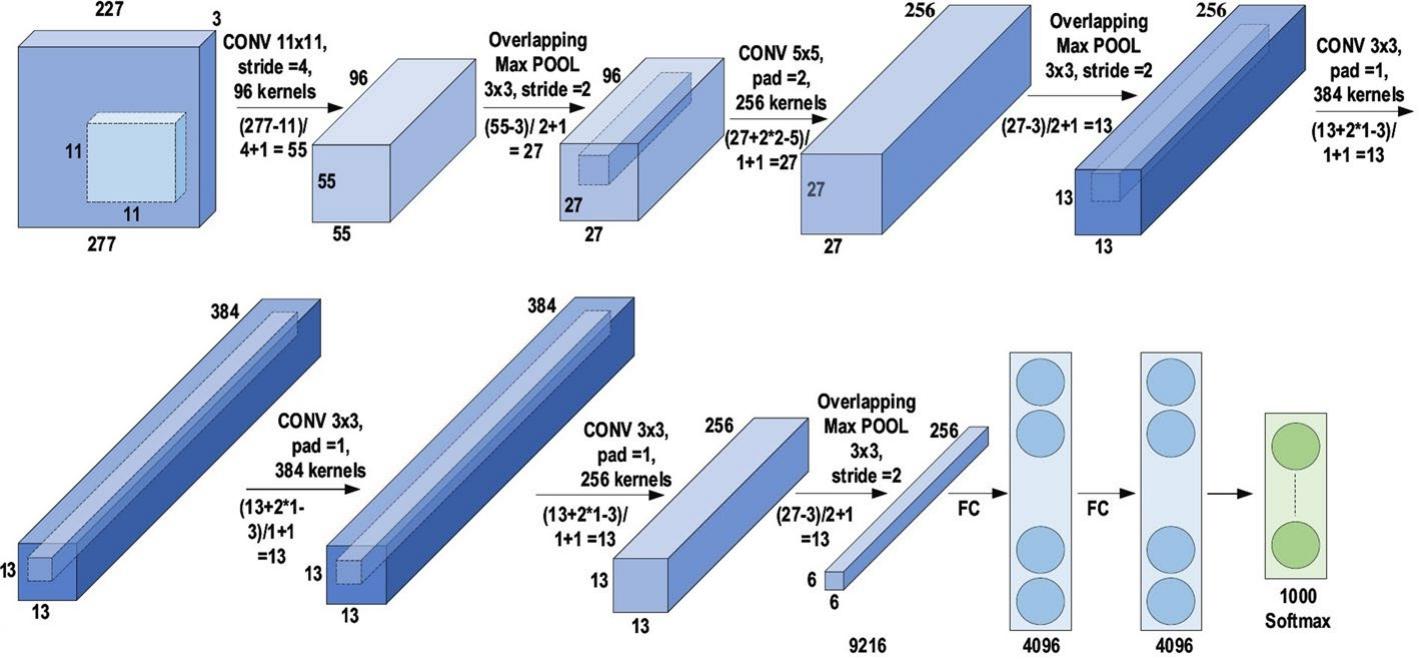
The architecture of AlexNet

The history of deep CNNs began with the appearance of LeNet [89] (Fig. 14). At that time, the CNNs were restricted to handwritten digit recognition tasks, which cannot be scaled to all image classes. In deep CNN architecture, AlexNet is highly respected [30], as it achieved innovative results in the fields of image recognition and classification. Krizhevesky et al. [30] first proposed AlexNet and consequently improved the CNN learning ability by increasing its depth and implementing several parameter optimization strategies. Figure 15 illustrates the basic design of the AlexNet architecture.

The learning ability of the deep CNN was limited at this time due to hardware restrictions. To overcome these hardware limitations, two GPUs (NVIDIA GTX 580) were used in parallel to train AlexNet. Moreover, in order to enhance the applicability of the CNN to different image categories, the number of feature extraction stages was increased from five in LeNet to seven in AlexNet. Regardless of the fact that depth enhances generalization for several image resolutions, it was in fact overfitting that represented the main drawback related to the depth. Krizhevesky et al. used Hinton’s idea to address this problem [90, 91]. To ensure that the features learned by the algorithm were extra robust, Krizhevesky et al.’s algorithm randomly passes over several transformational units throughout the training stage. Moreover, by reducing the vanishing gradient problem, ReLU [92] could be utilized as a non-saturating activation function to enhance the rate of convergence [93]. Local response normalization and overlapping subsampling were also performed to enhance the generalization by decreasing the overfitting. To improve on the performance of previous networks, other modifications were made by using large-size filters $(5\times 5 \; \text{and}\; 11 \times 11)$ in the earlier layers. AlexNet has considerable significance in the recent CNN generations, as well as beginning an innovative research era in CNN applications.

### Network-in-network

**Fig. 16:**
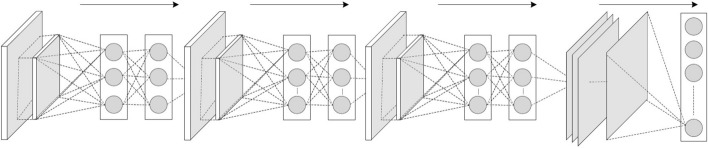
The architecture of network-in-network

This network model, which has some slight differences from the preceding models, introduced two innovative concepts [94]. The first was employing multiple layers of perception convolution. These convolutions are executed using a 1×1 filter, which supports the addition of extra nonlinearity in the networks. Moreover, this supports enlarging the network depth, which may later be regularized using dropout. For DL models, this idea is frequently employed in the bottleneck layer. As a substitution for a FC layer, the GAP is also employed, which represents the second novel concept and enables a significant reduction in the number of model parameters. In addition, GAP considerably updates the network architecture. Generating a final low-dimensional feature vector with no reduction in the feature maps dimension is possible when GAP is used on a large feature map [95, 96]. Figure 16 shows the structure of the network.

### ZefNet

**Fig. 17:**
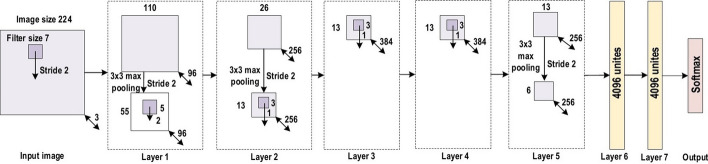
The architecture of ZefNet

Before 2013, the CNN learning mechanism was basically constructed on a trial-and-error basis, which precluded an understanding of the precise purpose following the enhancement. This issue restricted the deep CNN performance on convoluted images. In response, Zeiler and Fergus introduced DeconvNet (a multilayer de-convolutional neural network) in 2013 [97]. This method later became known as ZefNet, which was developed in order to quantitively visualize the network. Monitoring the CNN performance via understanding the neuron activation was the purpose of the network activity visualization. However, Erhan et al. utilized this exact concept to optimize deep belief network (DBN) performance by visualizing the features of the hidden layers [98]. Moreover, in addition to this issue, Le et al. assessed the deep unsupervised auto-encoder (AE) performance by visualizing the created classes of the image using the output neurons [99]. By reversing the operation order of the convolutional and pooling layers, DenconvNet operates like a forward-pass CNN. Reverse mapping of this kind launches the convolutional layer output backward to create visually observable image shapes that accordingly give the neural interpretation of the internal feature representation learned at each layer [100]. Monitoring the learning schematic through the training stage was the key concept underlying ZefNet. In addition, it utilized the outcomes to recognize an ability issue coupled with the model. This concept was experimentally proven on AlexNet by applying DeconvNet. This indicated that only certain neurons were working, while the others were out of action in the first two layers of the network. Furthermore, it indicated that the features extracted via the second layer contained aliasing objects. Thus, Zeiler and Fergus changed the CNN topology due to the existence of these outcomes. In addition, they executed parameter optimization, and also exploited the CNN learning by decreasing the stride and the filter sizes in order to retain all features of the initial two convolutional layers. An improvement in performance was accordingly achieved due to this rearrangement in CNN topology. This rearrangement proposed that the visualization of the features could be employed to identify design weaknesses and conduct appropriate parameter alteration. Figure 17 shows the structure of the network.

### Visual geometry group (VGG)

**Fig. 18:**
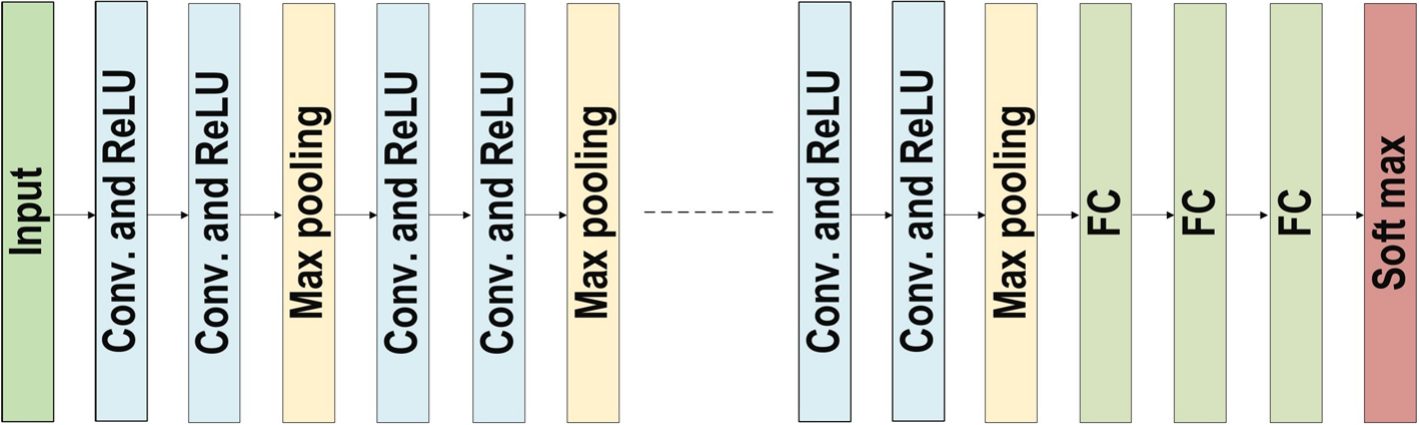
The architecture of VGG

After CNN was determined to be effective in the field of image recognition, an easy and efficient design principle for CNN was proposed by Simonyan and Zisserman. This innovative design was called Visual Geometry Group (VGG). A multilayer model [101], it featured nineteen more layers than ZefNet [97] and AlexNet [30] to simulate the relations of the network representational capacity in depth. Conversely, in the 2013-ILSVRC competition, ZefNet was the frontier network, which proposed that filters with small sizes could enhance the CNN performance. With reference to these results, VGG inserted a layer of the heap of $3\times 3$ filters rather than the $5\times 5$ and 11 × 11 filters in ZefNet. This showed experimentally that the parallel assignment of these small-size filters could produce the same influence as the large-size filters. In other words, these small-size filters made the receptive field similarly efficient to the large-size filters $(7 \times 7 \; \text{and}\; 5 \times 5)$. By decreasing the number of parameters, an extra advantage of reducing computational complication was achieved by using small-size filters. These outcomes established a novel research trend for working with small-size filters in CNN. In addition, by inserting $1\times 1$ convolutions in the middle of the convolutional layers, VGG regulates the network complexity. It learns a linear grouping of the subsequent feature maps. With respect to network tuning, a max pooling layer [102] is inserted following the convolutional layer, while padding is implemented to maintain the spatial resolution. In general, VGG obtained significant results for localization problems and image classification. While it did not achieve first place in the 2014-ILSVRC competition, it acquired a reputation due to its enlarged depth, homogenous topology, and simplicity. However, VGG’s computational cost was excessive due to its utilization of around 140 million parameters, which represented its main shortcoming. Figure 18 shows the structure of the network.

### GoogLeNet

**Fig. 19:**
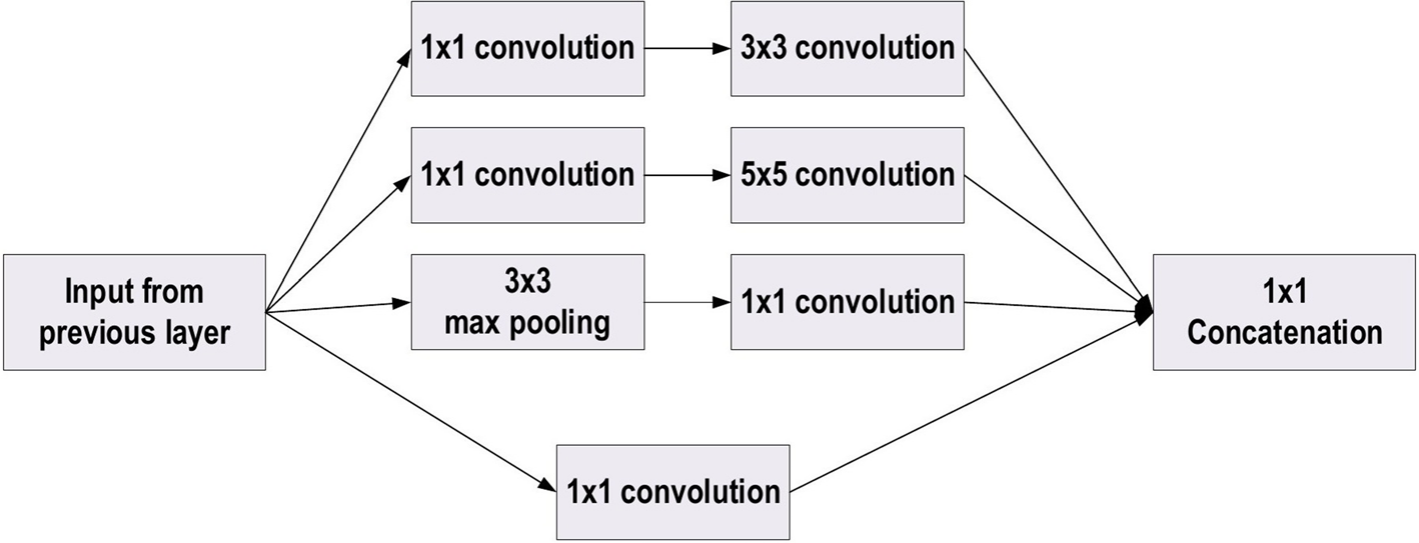
The basic structure of Google Block

In the 2014-ILSVRC competition, GoogleNet (also called Inception-V1) emerged as the winner [103]. Achieving high-level accuracy with decreased computational cost is the core aim of the GoogleNet architecture. It proposed a novel inception block (module) concept in the CNN context, since it combines multiple-scale convolutional transformations by employing merge, transform, and split functions for feature extraction. Figure 19 illustrates the inception block architecture. This architecture incorporates filters of different sizes ($5\times 5, 3\times 3, \; \text{and} \; 1\times 1$) to capture channel information together with spatial information at diverse ranges of spatial resolution. The common convolutional layer of GoogLeNet is substituted by small blocks using the same concept of network-in-network (NIN) architecture [94], which replaced each layer with a micro-neural network. The GoogLeNet concepts of merge, transform, and split were utilized, supported by attending to an issue correlated with different learning types of variants existing in a similar class of several images. The motivation of GoogLeNet was to improve the efficiency of CNN parameters, as well as to enhance the learning capacity. In addition, it regulates the computation by inserting a $1\times 1$ convolutional filter, as a bottleneck layer, ahead of using large-size kernels. GoogleNet employed sparse connections to overcome the redundant information problem. It decreased cost by neglecting the irrelevant channels. It should be noted here that only some of the input channels are connected to some of the output channels. By employing a GAP layer as the end layer, rather than utilizing a FC layer, the density of connections was decreased. The number of parameters was also significantly decreased from 40 to 5 million parameters due to these parameter tunings. The additional regularity factors used included the employment of RmsProp as optimizer and batch normalization [104]. Furthermore, GoogleNet proposed the idea of auxiliary learners to speed up the rate of convergence. Conversely, the main shortcoming of GoogleNet was its heterogeneous topology; this shortcoming requires adaptation from one module to another. Other shortcomings of GoogleNet include the representation jam, which substantially decreased the feature space in the following layer, and in turn occasionally leads to valuable information loss.

### Highway network

Increasing the network depth enhances its performance, mainly for complicated tasks. By contrast, the network training becomes difficult. The presence of several layers in deeper networks may result in small gradient values of the back-propagation of error at lower layers. In 2015, Srivastava et al. [105] suggested a novel CNN architecture, called Highway Network, to overcome this issue. This approach is based on the cross-connectivity concept. The unhindered information flow in Highway Network is empowered by instructing two gating units inside the layer. The gate mechanism concept was motivated by LSTM-based RNN [106, 107]. The information aggregation was conducted by merging the information of the $\i{\text{th}}-k$ layers with the next $\i{\text{th}}$ layer to generate a regularization impact, which makes the gradient-based training of the deeper network very simple. This empowers the training of networks with more than 100 layers, such as a deeper network of 900 layers with the SGD algorithm. A Highway Network with a depth of fifty layers presented an improved rate of convergence, which is better than thin and deep architectures at the same time [108]. By contrast, [69] empirically demonstrated that plain Net performance declines when more than ten hidden layers are inserted. It should be noted that even a Highway Network 900 layers in depth converges much more rapidly than the plain network.

### ResNet

**Fig. 20:**
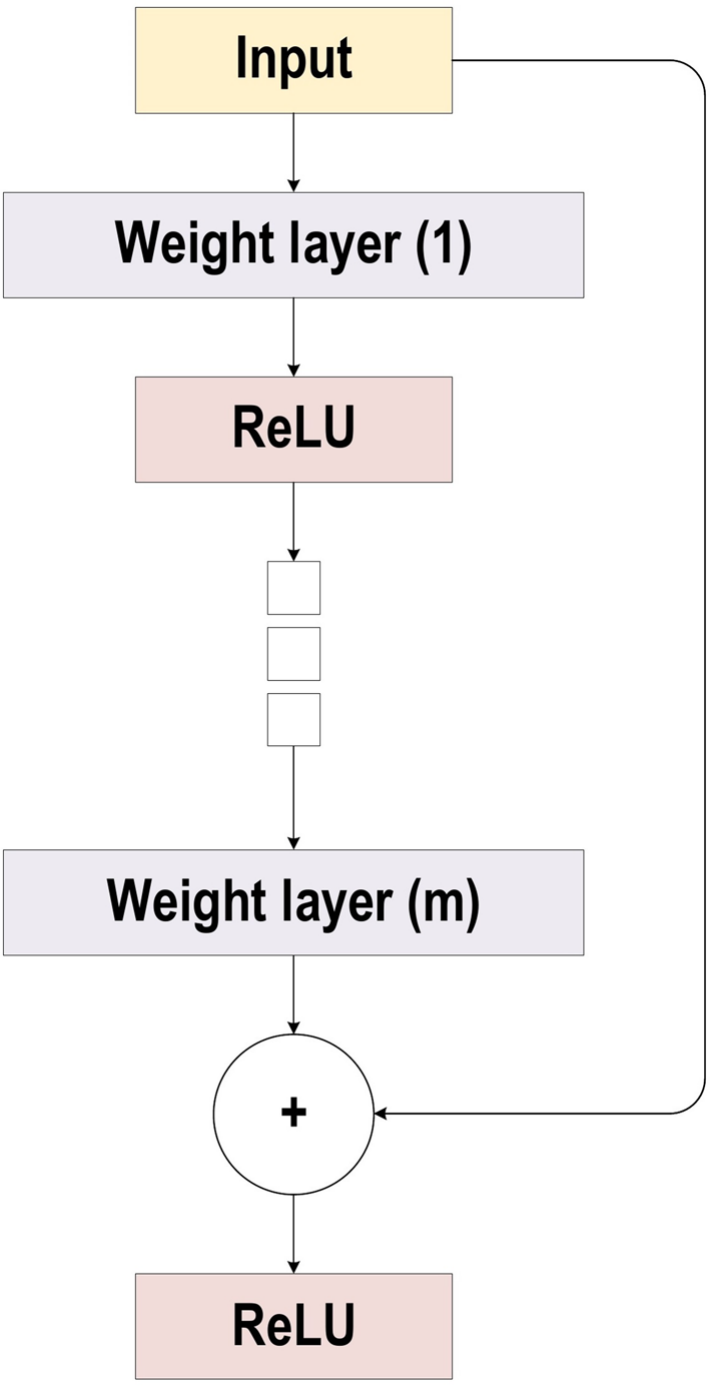
The block diagram for ResNet

He et al. [37] developed ResNet (Residual Network), which was the winner of ILSVRC 2015. Their objective was to design an ultra-deep network free of the vanishing gradient issue, as compared to the previous networks. Several types of ResNet were developed based on the number of layers (starting with 34 layers and going up to 1202 layers). The most common type was ResNet50, which comprised 49 convolutional layers plus a single FC layer. The overall number of network weights was 25.5 M, while the overall number of MACs was 3.9 M. The novel idea of ResNet is its use of the bypass pathway concept, as shown in Fig. 20, which was employed in Highway Nets to address the problem of training a deeper network in 2015. This is illustrated in Fig. 20, which contains the fundamental ResNet block diagram. This is a conventional feedforward network plus a residual connection. The residual layer output can be identified as the $(l - 1){\text{th}}$ outputs, which are delivered from the preceding layer $(x_{l} - 1)$. After executing different operations [such as convolution using variable-size filters, or batch normalization, before applying an activation function like ReLU on $(x_{l} - 1)$], the output is $F(x_{l} - 1)$. The ending residual output is $x_{l}$, which can be mathematically represented as in Eq. 18.

18 $$ x_{l}= F(x_{l} - 1)+x_{l} - 1 $$

There are numerous basic residual blocks included in the residual network. Based on the type of the residual network architecture, operations in the residual block are also changed [37].

In comparison to the highway network, ResNet presented shortcut connections inside layers to enable cross-layer connectivity, which are parameter-free and data-independent. Note that the layers characterize non-residual functions when a gated shortcut is closed in the highway network. By contrast, the individuality shortcuts are never closed, while the residual information is permanently passed in ResNet. Furthermore, ResNet has the potential to prevent the problems of gradient diminishing, as the shortcut connections (residual links) accelerate the deep network convergence. ResNet was the winner of the 2015-ILSVRC championship with 152 layers of depth; this represents 8 times the depth of VGG and 20 times the depth of AlexNet. In comparison with VGG, it has lower computational complexity, even with enlarged depth.

### Inception: ResNet and Inception-V3/4

**Fig. 21:**
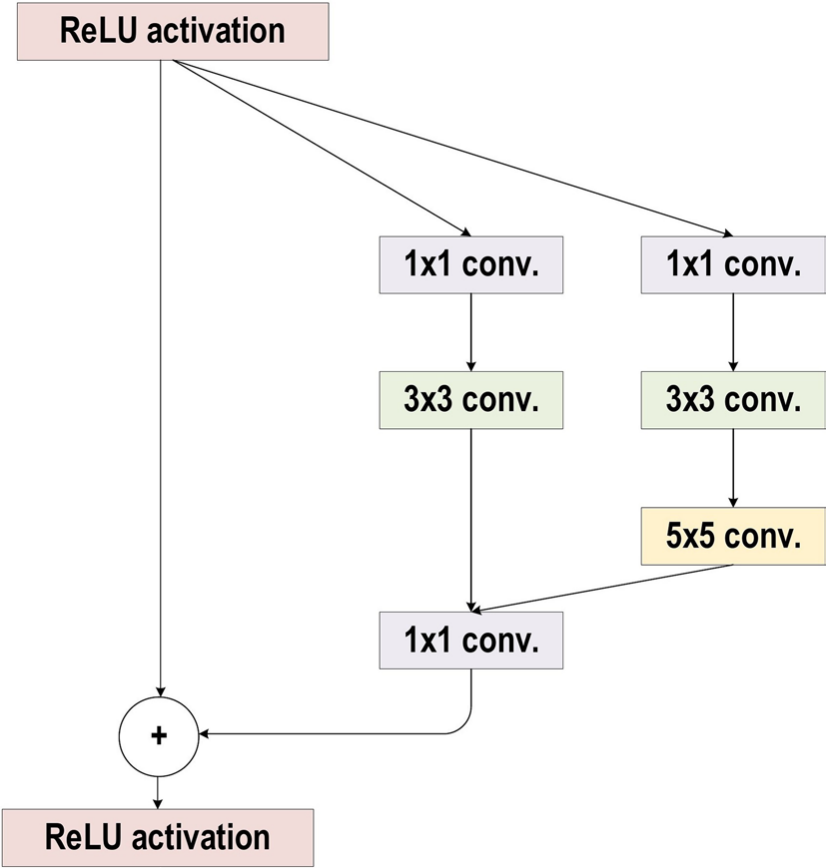
The basic block diagram for Inception Residual unit

Szegedy et al. [103, 109, 110] proposed Inception-ResNet and Inception-V3/4 as upgraded types of Inception-V1/2. The concept behind Inception-V3 was to minimize the computational cost with no effect on the deeper network generalization. Thus, Szegedy et al. used asymmetric small-size filters ($1\times 5$ and $1\times 7$) rather than large-size filters ($7\times 7$ and $5\times 5$); moreover, they utilized a bottleneck of $1\times 1$ convolution prior to the large-size filters [110]. These changes make the operation of the traditional convolution very similar to cross-channel correlation. Previously, Lin et al. utilized the 1 × 1 filter potential in NIN architecture [94]. Subsequently, [110] utilized the same idea in an intelligent manner. By using $1\times 1$ convolutional operation in Inception-V3, the input data are mapped into three or four isolated spaces, which are smaller than the initial input spaces. Next, all of these correlations are mapped in these smaller spaces through common $5\times 5$ or $3\times 3$ convolutions. By contrast, in Inception-ResNet, Szegedy et al. bring together the inception block and the residual learning power by replacing the filter concatenation with the residual connection [111]. Szegedy et al. empirically demonstrated that Inception-ResNet (Inception-4 with residual connections) can achieve a similar generalization power to Inception-V4 with enlarged width and depth and without residual connections. Thus, it is clearly illustrated that using residual connections in training will significantly accelerate the Inception network training. Figure 21 shows The basic block diagram for Inception Residual unit.

### DenseNet

**Fig. 22:**
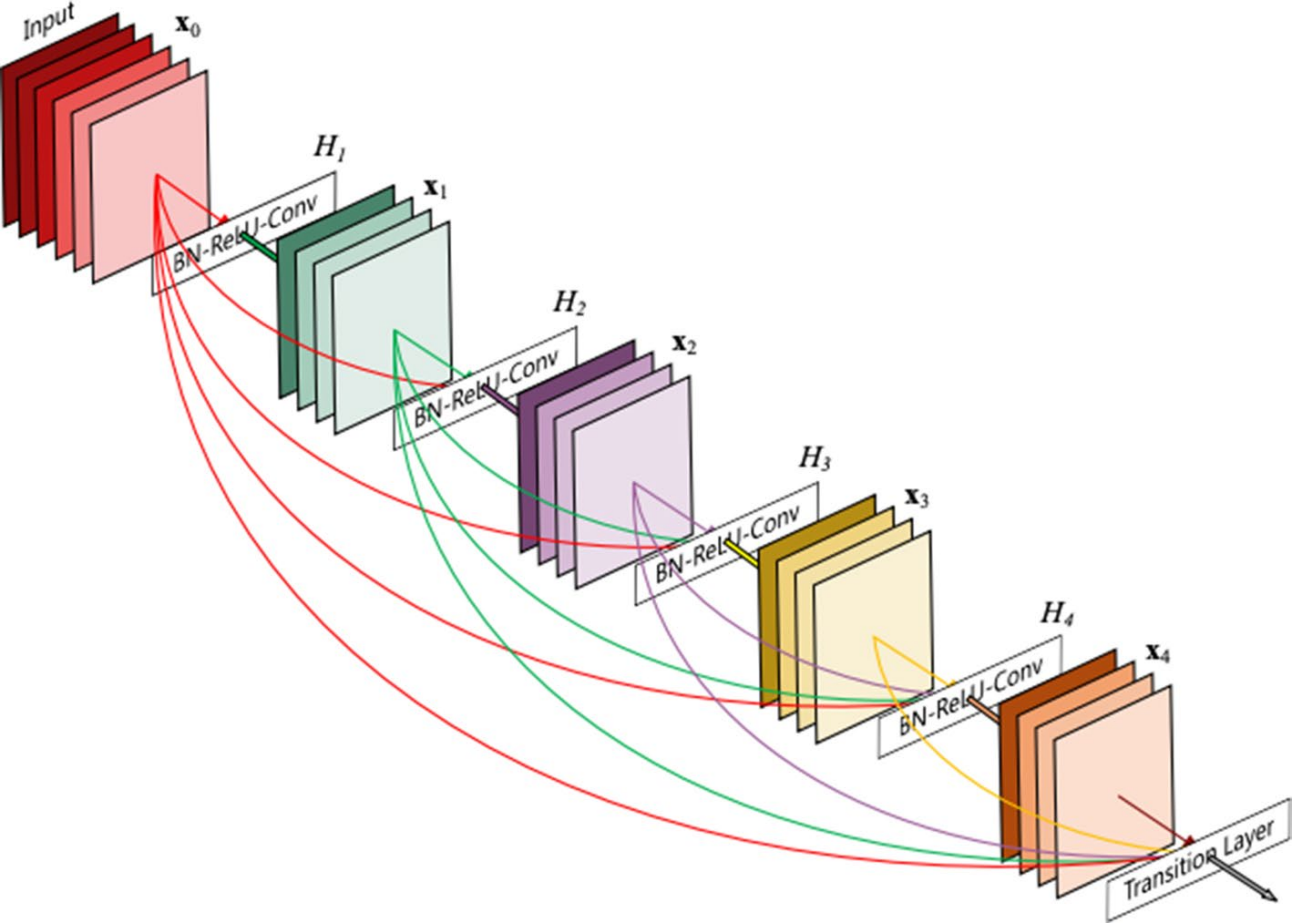
The architecture of DenseNet Network (adopted from [112])

To solve the problem of the vanishing gradient, DenseNet was presented, following the same direction as ResNet and the Highway network [105, 111, 112]. One of the drawbacks of ResNet is that it clearly conserves information by means of preservative individuality transformations, as several layers contribute extremely little or no information. In addition, ResNet has a large number of weights, since each layer has an isolated group of weights. DenseNet employed cross-layer connectivity in an improved approach to address this problem [112–114]. It connected each layer to all layers in the network using a feed-forward approach. Therefore, the feature maps of each previous layer were employed to input into all of the following layers. In traditional CNNs, there are *l* connections between the previous layer and the current layer, while in DenseNet, there are $\frac{l(l+1)}{2}$ direct connections. DenseNet demonstrates the influence of cross-layer depth wise-convolutions. Thus, the network gains the ability to discriminate clearly between the added and the preserved information, since DenseNet concatenates the features of the preceding layers rather than adding them. However, due to its narrow layer structure, DenseNet becomes parametrically high-priced in addition to the increased number of feature maps. The direct admission of all layers to the gradients via the loss function enhances the information flow all across the network. In addition, this includes a regularizing impact, which minimizes overfitting on tasks alongside minor training sets. Figure 22 shows the architecture of DenseNet Network.

### ResNext

**Fig. 23:**
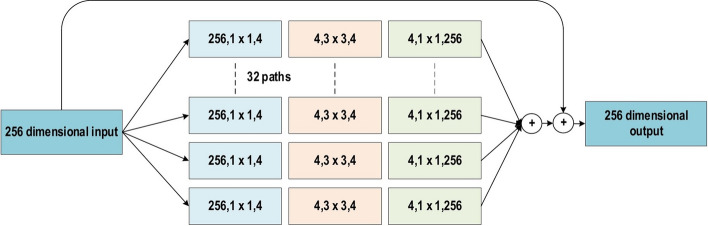
The basic block diagram for the ResNext building blocks

ResNext is an enhanced version of the Inception Network [115]. It is also known as the Aggregated Residual Transform Network. Cardinality, which is a new term presented by [115], utilized the split, transform, and merge topology in an easy and effective way. It denotes the size of the transformation set as an extra dimension [116–118]. However, the Inception network manages network resources more efficiently, as well as enhancing the learning ability of the conventional CNN. In the transformation branch, different spatial embeddings (employing e.g. $5\times 5$, $3\times 3$, and $1\times 1$) are used. Thus, customizing each layer is required separately. By contrast, ResNext derives its characteristic features from ResNet, VGG, and Inception. It employed the VGG deep homogenous topology with the basic architecture of GoogleNet by setting $3\times 3$ filters as spatial resolution inside the blocks of split, transform, and merge. Figure 23 shows the ResNext building blocks. ResNext utilized multi-transformations inside the blocks of split, transform, and merge, as well as outlining such transformations in cardinality terms. The performance is significantly improved by increasing the cardinality, as Xie et al. showed. The complexity of ResNext was regulated by employing $1\times 1$ filters (low embeddings) ahead of a $3\times 3$ convolution. By contrast, skipping connections are used for optimized training [115].

### WideResNet

The feature reuse problem is the core shortcoming related to deep residual networks, since certain feature blocks or transformations contribute a very small amount to learning. Zagoruyko and Komodakis [119] accordingly proposed WideResNet to address this problem. These authors advised that the depth has a supplemental influence, while the residual units convey the core learning ability of deep residual networks. WideResNet utilized the residual block power via making the ResNet wider instead of deeper [37]. It enlarged the width by presenting an extra factor, k, which handles the network width. In other words, it indicated that layer widening is a highly successful method of performance enhancement compared to deepening the residual network. While enhanced representational capacity is achieved by deep residual networks, these networks also have certain drawbacks, such as the exploding and vanishing gradient problems, feature reuse problem (inactivation of several feature maps), and the time-intensive nature of the training. He et al. [37] tackled the feature reuse problem by including a dropout in each residual block to regularize the network in an efficient manner. In a similar manner, utilizing dropouts, Huang et al. [120] presented the stochastic depth concept to solve the slow learning and gradient vanishing problems. Earlier research was focused on increasing the depth; thus, any small enhancement in performance required the addition of several new layers. When comparing the number of parameters, WideResNet has twice that of ResNet, as an experimental study showed. By contrast, WideResNet presents an improved method for training relative to deep networks [119]. Note that most architectures prior to residual networks (including the highly effective VGG and Inception) were wider than ResNet. Thus, wider residual networks were established once this was determined. However, inserting a dropout between the convolutional layers (as opposed to within the residual block) made the learning more effective in WideResNet [121, 122].

### Pyramidal Net

The depth of the feature map increases in the succeeding layer due to the deep stacking of multi-convolutional layers, as shown in previous deep CNN architectures such as ResNet, VGG, and AlexNet. By contrast, the spatial dimension reduces, since a sub-sampling follows each convolutional layer. Thus, augmented feature representation is recompensed by decreasing the size of the feature map. The extreme expansion in the depth of the feature map, alongside the spatial information loss, interferes with the learning ability in the deep CNNs. ResNet obtained notable outcomes for the issue of image classification. Conversely, deleting a convolutional block—in which both the number of channel and spatial dimensions vary (channel depth enlarges, while spatial dimension reduces)—commonly results in decreased classifier performance. Accordingly, the stochastic ResNet enhanced the performance by decreasing the information loss accompanying the residual unit drop. Han et al. [123] proposed Pyramidal Net to address the ResNet learning interference problem. To address the depth enlargement and extreme reduction in spatial width via ResNet, Pyramidal Net slowly enlarges the residual unit width to cover the most feasible places rather than saving the same spatial dimension inside all residual blocks up to the appearance of the down-sampling. It was referred to as Pyramidal Net due to the slow enlargement in the feature map depth based on the up-down method. Factor l, which was determined by Eq. 19, regulates the depth of the feature map.

19 $$\begin{aligned} d_{l}=\begin{Bmatrix} 16&if \,\, l =1\\ \left\lfloor d_{l-1}+\frac{\lambda }{n} \right\rfloor&if \,\,2 \le l \le n+1 \end{Bmatrix} \end{aligned}$$

Here, the dimension of the *l*th residual unit is indicated by $d_{l}$; moreover, *n* indicates the overall number of residual units, the step factor is indicated by $\lambda$, and the depth increase is regulated by the factor $\frac{\lambda }{n}$, which uniformly distributes the weight increase across the dimension of the feature map. Zero-padded identity mapping is used to insert the residual connections among the layers. In comparison to the projection-based shortcut connections, zero-padded identity mapping requires fewer parameters, which in turn leads to enhanced generalization [124]. Multiplication- and addition-based widening are two different approaches used in Pyramidal Nets for network widening. More specifically, the first approach (multiplication) enlarges geometrically, while the second one (addition) enlarges linearly [92]. The main problem associated with the width enlargement is the growth in time and space required related to the quadratic time.

### Xception

**Fig. 24:**
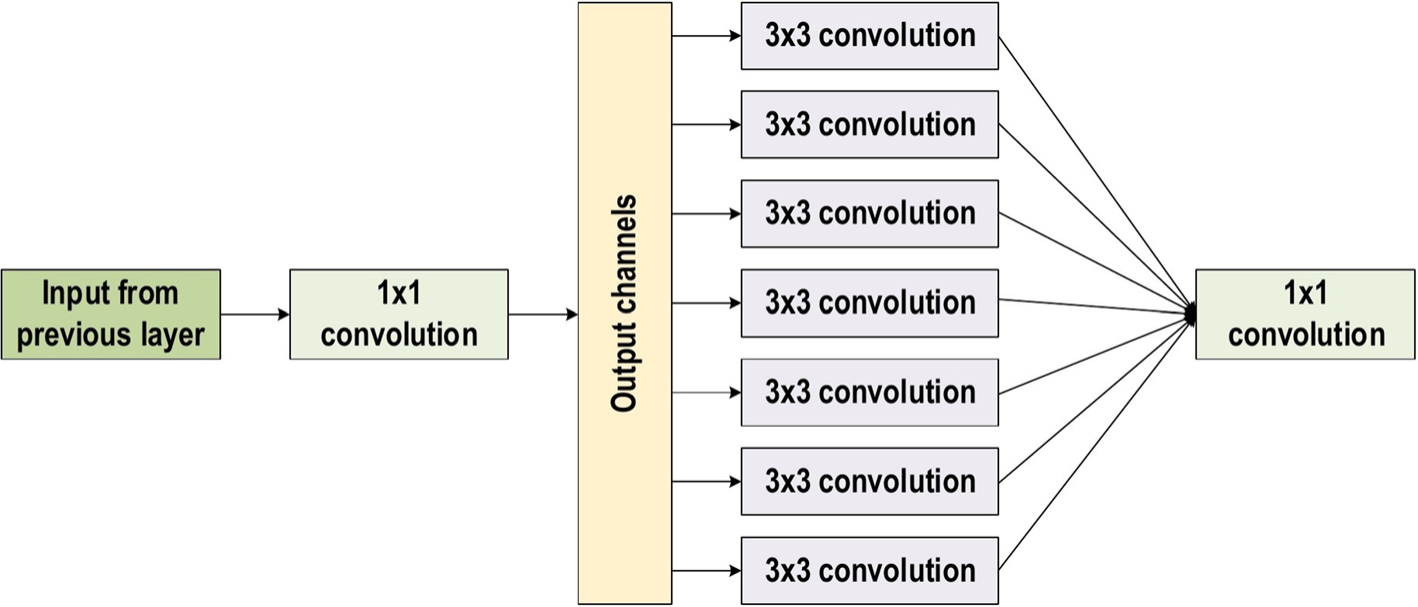
The basic block diagram for the Xception block architecture

Extreme inception architecture is the main characteristic of Xception. The main idea behind Xception is its depthwise separable convolution [125]. The Xception model adjusted the original inception block by making it wider and exchanging a single dimension ($3 \times 3$) followed by a $1 \times 1$ convolution to reduce computational complexity. Figure 24 shows the Xception block architecture. The Xception network becomes extra computationally effective through the use of the decoupling channel and spatial correspondence. Moreover, it first performs mapping of the convolved output to the embedding short dimension by applying $1 \times 1$ convolutions. It then performs *k* spatial transformations. Note that *k* here represents the width-defining cardinality, which is obtained via the transformations number in Xception. However, the computations were made simpler in Xception by distinctly convolving each channel around the spatial axes. These axes are subsequently used as the $1 \times 1$ convolutions (pointwise convolution) for performing cross-channel correspondence. The $1 \times 1$ convolution is utilized in Xception to regularize the depth of the channel. The traditional convolutional operation in Xception utilizes a number of transformation segments equivalent to the number of channels; Inception, moreover, utilizes three transformation segments, while traditional CNN architecture utilizes only a single transformation segment. Conversely, the suggested Xception transformation approach achieves extra learning efficiency and better performance but does not minimize the number of parameters [126, 127].

### Residual attention neural network

To improve the network feature representation, Wang et al. [128] proposed the Residual Attention Network (RAN). Enabling the network to learn aware features of the object is the main purpose of incorporating attention into the CNN. The RAN consists of stacked residual blocks in addition to the attention module; hence, it is a feed-forward CNN. However, the attention module is divided into two branches, namely the mask branch and trunk branch. These branches adopt a top-down and bottom-up learning strategy respectively. Encapsulating two different strategies in the attention model supports top-down attention feedback and fast feed-forward processing in only one particular feed-forward process. More specifically, the top-down architecture generates dense features to make inferences about every aspect. Moreover, the bottom-up feedforward architecture generates low-resolution feature maps in addition to robust semantic information. Restricted Boltzmann machines employed a top-down bottom-up strategy as in previously proposed studies [129]. During the training reconstruction phase, Goh et al. [130] used the mechanism of top-down attention in deep Boltzmann machines (DBMs) as a regularizing factor. Note that the network can be globally optimized using a top-down learning strategy in a similar manner, where the maps progressively output to the input throughout the learning process [129–132].

Incorporating the attention concept with convolutional blocks in an easy way was used by the transformation network, as obtained in a previous study [133]. Unfortunately, these are inflexible, which represents the main problem, along with their inability to be used for varying surroundings. By contrast, stacking multi-attention modules has made RAN very effective at recognizing noisy, complex, and cluttered images. RAN’s hierarchical organization gives it the capability to adaptively allocate a weight for every feature map depending on its importance within the layers. Furthermore, incorporating three distinct levels of attention (spatial, channel, and mixed) enables the model to use this ability to capture the object-aware features at these distinct levels.

### Convolutional block attention module

The importance of the feature map utilization and the attention mechanism is certified via SE-Network and RAN [128, 134, 135]. The convolutional block attention (CBAM) module, which is a novel attention-based CNN, was first developed by Woo et al. [136]. This module is similar to SE-Network and simple in design. SE-Network disregards the object’s spatial locality in the image and considers only the channels’ contribution during the image classification. Regarding object detection, object spatial location plays a significant role. The convolutional block attention module sequentially infers the attention maps. More specifically, it applies channel attention preceding the spatial attention to obtain the refined feature maps. Spatial attention is performed using 1 × 1 convolution and pooling functions, as in the literature. Generating an effective feature descriptor can be achieved by using a spatial axis along with the pooling of features. In addition, generating a robust spatial attention map is possible, as CBAM concatenates the max pooling and average pooling operations. In a similar manner, a collection of GAP and max pooling operations is used to model the feature map statistics. Woo et al. [136] demonstrated that utilizing GAP will return a sub-optimized inference of channel attention, whereas max pooling provides an indication of the distinguishing object features. Thus, the utilization of max pooling and average pooling enhances the network’s representational power. The feature maps improve the representational power, as well as facilitating a focus on the significant portion of the chosen features. The expression of 3D attention maps through a serial learning procedure assists in decreasing the computational cost and the number of parameters, as Woo et al. [136] experimentally proved. Note that any CNN architecture can be simply integrated with CBAM.

### Concurrent spatial and channel excitation mechanism

To make the work valid for segmentation tasks, Roy et al. [137, 138] expanded Hu et al. [134] effort by adding the influence of spatial information to the channel information. Roy et al. [137, 138] presented three types of modules: (1) channel squeeze and excitation with concurrent channels (scSE); (2) exciting spatially and squeezing channel-wise (sSE); (3) exciting channel-wise and squeezing spatially (cSE). For segmentation purposes, they employed auto-encoder-based CNNs. In addition, they suggested inserting modules following the encoder and decoder layers. To specifically highlight the object-specific feature maps, they further allocated attention to every channel by expressing a scaling factor from the channel and spatial information in the first module (scSE). In the second module (sSE), the feature map information has lower importance than the spatial locality, as the spatial information plays a significant role during the segmentation process. Therefore, several channel collections are spatially divided and developed so that they can be employed in segmentation. In the final module (cSE), a similar SE-block concept is used. Furthermore, the scaling factor is derived founded on the contribution of the feature maps within the object detection [137, 138].

### CapsuleNet

CNN is an efficient technique for detecting object features and achieving well-behaved recognition performance in comparison with innovative handcrafted feature detectors. A number of restrictions related to CNN are present, meaning that the CNN does not consider certain relations, orientation, size, and perspectives of features. For instance, when considering a face image, the CNN does not count the various face components (such as mouth, eyes, nose, etc.) positions, and will incorrectly activate the CNN neurons and recognize the face without taking specific relations (such as size, orientation etc.) into account. At this point, consider a neuron that has probability in addition to feature properties such as size, orientation, perspective, etc. A specific neuron/capsule of this type has the ability to effectively detect the face along with different types of information. Thus, many layers of capsule nodes are used to construct the capsule network. An encoding unit, which contains three layers of capsule nodes, forms the CapsuleNet or CapsNet (the initial version of the capsule networks).

For example, the MNIST architecture comprises $28\times 28$ images, applying 256 filters of size $9\times 9$ and with stride 1. The $28-9+1=20$ is the output plus 256 feature maps. Next, these outputs are input to the first capsule layer, while producing an 8D vector rather than a scalar; in fact, this is a modified convolution layer. Note that a stride 2 with $9\times 9$ filters is employed in the first convolution layer. Thus, the dimension of the output is $(20-9)/2+1=6$. The initial capsules employ $8\times 32$ filters, which generate 32 × 8 × 6 × 6 (32 for groups, 8 for neurons, while 6 × 6 is the neuron size).

**Fig. 25:**
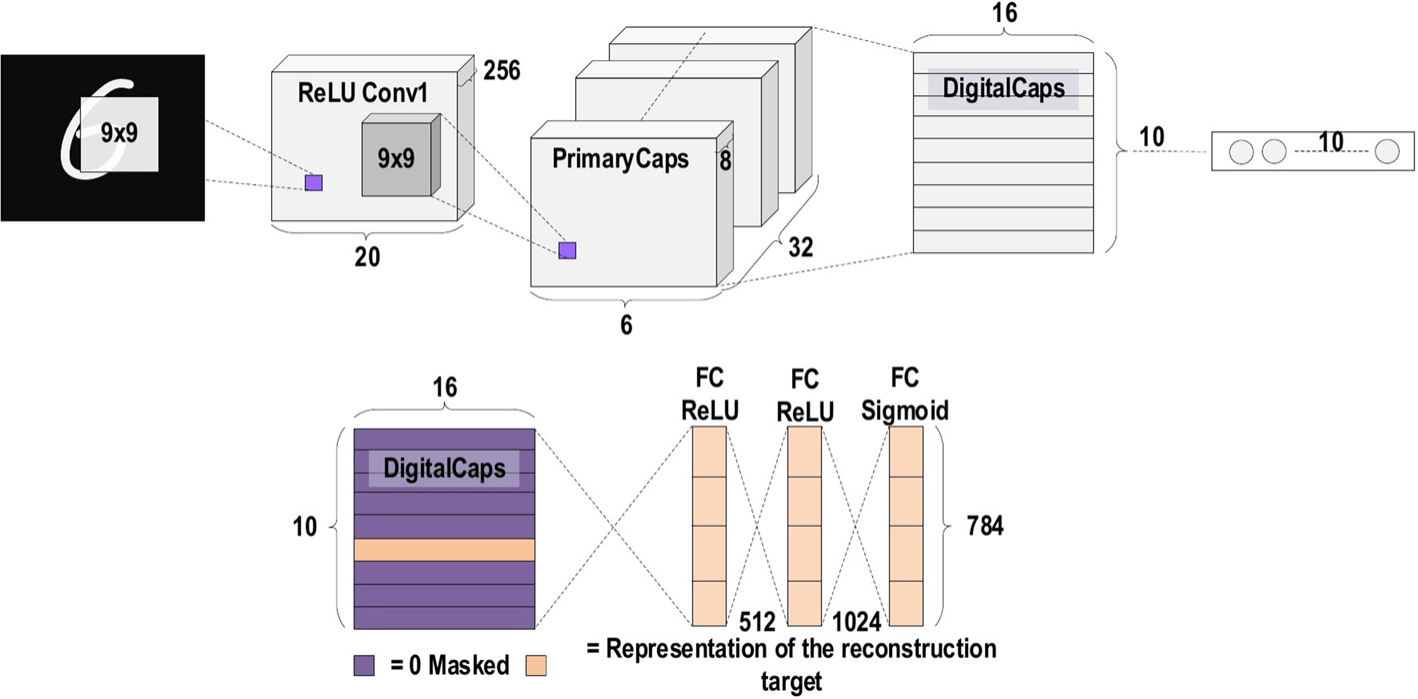
The complete CapsNet encoding and decoding processes

Figure 25 represents the complete CapsNet encoding and decoding processes. In the CNN context, a max-pooling layer is frequently employed to handle the translation change. It can detect the feature moves in the event that the feature is still within the max-pooling window. This approach has the ability to detect the overlapped features; this is highly significant in detection and segmentation operations, since the capsule involves the weighted features sum from the preceding layer.

In conventional CNNs, a particular cost function is employed to evaluate the global error that grows toward the back throughout the training process. Conversely, in such cases, the activation of a neuron will not grow further once the weight between two neurons turns out to be zero. Instead of a single size being provided with the complete cost function in repetitive dynamic routing alongside the agreement, the signal is directed based on the feature parameters. Sabour et al. [139] provides more details about this architecture. When using MNIST to recognize handwritten digits, this innovative CNN architecture gives superior accuracy. From the application perspective, this architecture has extra suitability for segmentation and detection approaches when compared with classification approaches [140–142].

### High-resolution network (HRNet)

**Fig. 26:**
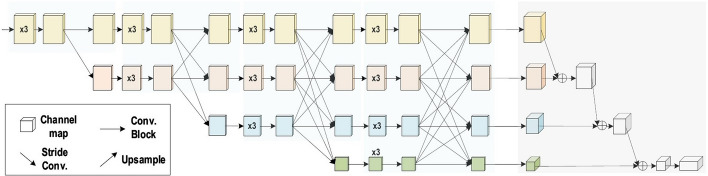
The general architecture of HRNet

High-resolution representations are necessary for position-sensitive vision tasks, such as semantic segmentation, object detection, and human pose estimation. In the present up-to-date frameworks, the input image is encoded as a low-resolution representation using a subnetwork that is constructed as a connected series of high-to-low resolution convolutions such as VGGNet and ResNet. The low-resolution representation is then recovered to become a high-resolution one. Alternatively, high-resolution representations are maintained during the entire process using a novel network, referred to as a High-Resolution Network (HRNet) [143, 144]. This network has two principal features. First, the convolution series of high-to-low resolutions are connected in parallel. Second, the information across the resolutions are repeatedly exchanged. The advantage achieved includes getting a representation that is more accurate in the spatial domain and extra-rich in the semantic domain. Moreover, HRNet has several applications in the fields of object detection, semantic segmentation, and human pose prediction. For computer vision problems, the HRNet represents a more robust backbone. Figure 26 illustrates the general architecture of HRNet.

## Challenges (limitations) of deep learning and alternate solutions

When employing DL, several difficulties are often taken into consideration. Those more challenging are listed next and several possible alternatives are accordingly provided.

### Training data

**Fig. 27:**
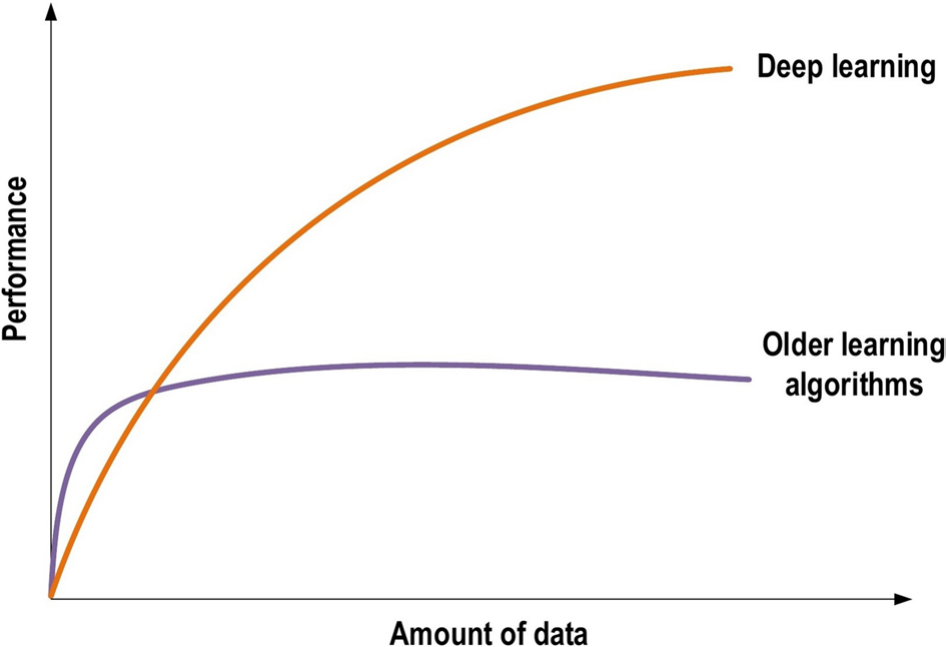
The performance of DL regarding the amount of data

DL is extremely data-hungry considering it also involves representation learning [145, 146]. DL demands an extensively large amount of data to achieve a well-behaved performance model, i.e. as the data increases, an extra well-behaved performance model can be achieved (Fig. 27). In most cases, the available data are sufficient to obtain a good performance model. However, sometimes there is a shortage of data for using DL directly [87]. To properly address this issue, three suggested methods are available. The first involves the employment of the transfer-learning concept after data is collected from similar tasks. Note that while the transferred data will not directly augment the actual data, it will help in terms of both enhancing the original input representation of data and its mapping function [147]. In this way, the model performance is boosted. Another technique involves employing a well-trained model from a similar task and fine-tuning the ending of two layers or even one layer based on the limited original data. Refer to [148, 149] for a review of different transfer-learning techniques applied in the DL approach. In the second method, data augmentation is performed [150]. This task is very helpful for use in augmenting the image data, since the image translation, mirroring, and rotation commonly do not change the image label. Conversely, it is important to take care when applying this technique in some cases such as with bioinformatics data. For instance, when mirroring an enzyme sequence, the output data may not represent the actual enzyme sequence. In the third method, the simulated data can be considered for increasing the volume of the training set. It is occasionally possible to create simulators based on the physical process if the issue is well understood. Therefore, the result will involve the simulation of as much data as needed. Processing the data requirement for DL-based simulation is obtained as an example in Ref. [151].

#### Transfer learning

Recent research has revealed a widespread use of deep CNNs, which offer ground-breaking support for answering many classification problems. Generally speaking, deep CNN models require a sizable volume of data to obtain good performance. The common challenge associated with using such models concerns the lack of training data. Indeed, gathering a large volume of data is an exhausting job, and no successful solution is available at this time. The undersized dataset problem is therefore currently solved using the TL technique [148, 149], which is highly efficient in addressing the lack of training data issue. The mechanism of TL involves training the CNN model with large volumes of data. In the next step, the model is fine-tuned for training on a small request dataset.

**Fig. 28:**
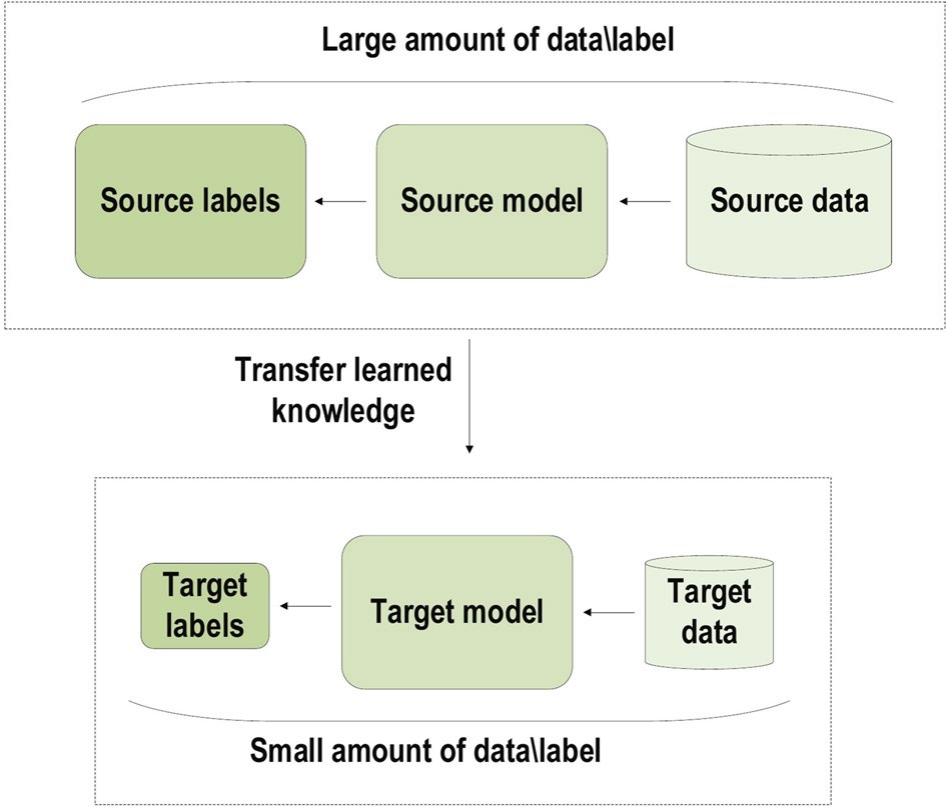
The conceptual diagram of the TL technique

The student-teacher relationship is a suitable approach to clarifying TL. Gathering detailed knowledge of the subject is the first step [152]. Next, the teacher provides a “course” by conveying the information within a “lecture series” over time. Put simply, the teacher transfers the information to the student. In more detail, the expert (teacher) transfers the knowledge (information) to the learner (student). Similarly, the DL network is trained using a vast volume of data, and also learns the bias and the weights during the training process. These weights are then transferred to different networks for retraining or testing a similar novel model. Thus, the novel model is enabled to pre-train weights rather than requiring training from scratch. Figure 28 illustrates the conceptual diagram of the TL technique.

1. Pre-trained models: Many CNN models, e.g. AlexNet [30], GoogleNet [103], and ResNet [37], have been trained on large datasets such as ImageNet for image recognition purposes. These models can then be employed to recognize a different task without the need to train from scratch. Furthermore, the weights remain the same apart from a few learned features. In cases where data samples are lacking, these models are very useful. There are many reasons for employing a pre-trained model. First, training large models on sizeable datasets requires high-priced computational power. Second, training large models can be time-consuming, taking up to multiple weeks. Finally, a pre-trained model can assist with network generalization and speed up the convergence.
2. A research problem using pre-trained models: Training a DL approach requires a massive number of images. Thus, obtaining good performance is a challenge under these circumstances. Achieving excellent outcomes in image classification or recognition applications, with performance occasionally superior to that of a human, becomes possible through the use of deep convolutional neural networks (DCNNs) including several layers if a huge amount of data is available [37, 148, 153]. However, avoiding overfitting problems in such applications requires sizable datasets and properly generalizing DCNN models. When training a DCNN model, the dataset size has no lower limit. However, the accuracy of the model becomes insufficient in the case of the utilized model has fewer layers, or if a small dataset is used for training due to over- or under-fitting problems. Due to they have no ability to utilize the hierarchical features of sizable datasets, models with fewer layers have poor accuracy. It is difficult to acquire sufficient training data for DL models. For example, in medical imaging and environmental science, gathering labelled datasets is very costly [148]. Moreover, the majority of the crowdsourcing workers are unable to make accurate notes on medical or biological images due to their lack of medical or biological knowledge. Thus, ML researchers often rely on field experts to label such images; however, this process is costly and time consuming. Therefore, producing the large volume of labels required to develop flourishing deep networks turns out to be unfeasible. Recently, TL has been widely employed to address the later issue. Nevertheless, although TL enhances the accuracy of several tasks in the fields of pattern recognition and computer vision [154, 155], there is an essential issue related to the source data type used by the TL as compared to the target dataset. For instance, enhancing the medical image classification performance of CNN models is achieved by training the models using the ImageNet dataset, which contains natural images [153]. However, such natural images are completely dissimilar from the raw medical images, meaning that the model performance is not enhanced. It has further been proven that TL from different domains does not significantly affect performance on medical imaging tasks, as lightweight models trained from scratch perform nearly as well as standard ImageNet-transferred models [156]. Therefore, there exists scenarios in which using pre-trained models do not become an affordable solution. In 2020, some researchers have utilized same-domain TL and achieved excellent results [86–88, 157]. Same-domain TL is an approach of using images that look similar to the target dataset for training. For example, using X-ray images of different chest diseases to train the model, then fine-tuning and training it on chest X-ray images for COVID-19 diagnosis. More details about same-domain TL and how to implement the fine-tuning process can be found in [87].

#### Data augmentation techniques

If the goal is to increase the amount of available data and avoid the overfitting issue, data augmentation techniques are one possible solution [150, 158, 159]. These techniques are data-space solutions for any limited-data problem. Data augmentation incorporates a collection of methods that improve the attributes and size of training datasets. Thus, DL networks can perform better when these techniques are employed. Next, we list some data augmentation alternate solutions.

1. Flipping: Flipping the vertical axis is a less common practice than flipping the horizontal one. Flipping has been verified as valuable on datasets like ImageNet and CIFAR-10. Moreover, it is highly simple to implement. In addition, it is not a label-conserving transformation on datasets that involve text recognition (such as SVHN and MNIST).
2. Color space: Encoding digital image data is commonly used as a dimension tensor ($height \times width \times color channels$). Accomplishing augmentations in the color space of the channels is an alternative technique, which is extremely workable for implementation. A very easy color augmentation involves separating a channel of a particular color, such as Red, Green, or Blue. A simple way to rapidly convert an image using a single-color channel is achieved by separating that matrix and inserting additional double zeros from the remaining two color channels. Furthermore, increasing or decreasing the image brightness is achieved by using straightforward matrix operations to easily manipulate the RGB values. By deriving a color histogram that describes the image, additional improved color augmentations can be obtained. Lighting alterations are also made possible by adjusting the intensity values in histograms similar to those employed in photo-editing applications.
3. Cropping: Cropping a dominant patch of every single image is a technique employed with combined dimensions of height and width as a specific processing step for image data. Furthermore, random cropping may be employed to produce an impact similar to translations. The difference between translations and random cropping is that translations conserve the spatial dimensions of this image, while random cropping reduces the input size [for example from (256, 256) to (224, 224)]. According to the selected reduction threshold for cropping, the label-preserving transformation may not be addressed.
4. Rotation: When rotating an image left or right from within 0 to 360 degrees around the axis, rotation augmentations are obtained. The rotation degree parameter greatly determines the suitability of the rotation augmentations. In digit recognition tasks, small rotations (from 0 to 20 degrees) are very helpful. By contrast, the data label cannot be preserved post-transformation when the rotation degree increases.
5. Translation: To avoid positional bias within the image data, a very useful transformation is to shift the image up, down, left, or right. For instance, it is common that the whole dataset images are centered; moreover, the tested dataset should be entirely made up of centered images to test the model. Note that when translating the initial images in a particular direction, the residual space should be filled with Gaussian or random noise, or a constant value such as 255 s or 0 s. The spatial dimensions of the image post-augmentation are preserved using this padding.
6. Noise injection This approach involves injecting a matrix of arbitrary values. Such a matrix is commonly obtained from a Gaussian distribution. Moreno-Barea et al. [160] employed nine datasets to test the noise injection. These datasets were taken from the UCI repository [161]. Injecting noise within images enables the CNN to learn additional robust features.
  However, highly well-behaved solutions for positional biases available within the training data are achieved by means of geometric transformations. To separate the distribution of the testing data from the training data, several prospective sources of bias exist. For instance, when all faces should be completely centered within the frames (as in facial recognition datasets), the problem of positional biases emerges. Thus, geometric translations are the best solution. Geometric translations are helpful due to their simplicity of implementation, as well as their effective capability to disable the positional biases. Several libraries of image processing are available, which enables beginning with simple operations such as rotation or horizontal flipping. Additional training time, higher computational costs, and additional memory are some shortcomings of geometric transformations. Furthermore, a number of geometric transformations (such as arbitrary cropping or translation) should be manually observed to ensure that they do not change the image label. Finally, the biases that separate the test data from the training data are more complicated than transitional and positional changes. Hence, it is not trivial answering to when and where geometric transformations are suitable to be applied.

### Imbalanced data

Commonly, biological data tend to be imbalanced, as negative samples are much more numerous than positive ones [162–164]. For example, compared to COVID-19-positive X-ray images, the volume of normal X-ray images is very large. It should be noted that undesirable results may be produced when training a DL model using imbalanced data. The following techniques are used to solve this issue. First, it is necessary to employ the correct criteria for evaluating the loss, as well as the prediction result. In considering the imbalanced data, the model should perform well on small classes as well as larger ones. Thus, the model should employ area under curve (AUC) as the resultant loss as well as the criteria [165]. Second, it should employ the weighted cross-entropy loss, which ensures the model will perform well with small classes if it still prefers to employ the cross-entropy loss. Simultaneously, during model training, it is possible either to down-sample the large classes or up-sample the small classes. Finally, to make the data balanced as in Ref. [166], it is possible to construct models for every hierarchical level, as a biological system frequently has hierarchical label space. However, the effect of the imbalanced data on the performance of the DL model has been comprehensively investigated. In addition, to lessen the problem, the most frequently used techniques were also compared. Nevertheless, note that these techniques are not specified for biological problems.

### Interpretability of data

Occasionally, DL techniques are analyzed to act as a black box. In fact, they are interpretable. The need for a method of interpreting DL, which is used to obtain the valuable motifs and patterns recognized by the network, is common in many fields, such as bioinformatics [167]. In the task of disease diagnosis, it is not only required to know the disease diagnosis or prediction results of a trained DL model, but also how to enhance the surety of the prediction outcomes, as the model makes its decisions based on these verifications [168]. To achieve this, it is possible to give a score of importance for every portion of the particular example. Within this solution, back-propagation-based techniques or perturbation-based approaches are used [169]. In the perturbation-based approaches, a portion of the input is changed and the effect of this change on the model output is observed [170–173]. This concept has high computational complexity, but it is simple to understand. On the other hand, to check the score of the importance of various input portions, the signal from the output propagates back to the input layer in the back-propagation-based techniques. These techniques have been proven valuable in [174]. In different scenarios, various meanings can represent the model interpretability.

### Uncertainty scaling

Commonly, the final prediction label is not the only label required when employing DL techniques to achieve the prediction; the score of confidence for every inquiry from the model is also desired. The score of confidence is defined as how confident the model is in its prediction [175]. Since the score of confidence prevents belief in unreliable and misleading predictions, it is a significant attribute, regardless of the application scenario. In biology, the confidence score reduces the resources and time expended in proving the outcomes of the misleading prediction. Generally speaking, in healthcare or similar applications, the uncertainty scaling is frequently very significant; it helps in evaluating automated clinical decisions and the reliability of machine learning-based disease-diagnosis [176, 177]. Because overconfident prediction can be the output of different DL models, the score of probability (achieved from the softmax output of the direct-DL) is often not in the correct scale [178]. Note that the softmax output requires post-scaling to achieve a reliable probability score. For outputting the probability score in the correct scale, several techniques have been introduced, including Bayesian Binning into Quantiles (BBQ) [179], isotonic regression [180], histogram binning [181], and the legendary Platt scaling [182]. More specifically, for DL techniques, temperature scaling was recently introduced, which achieves superior performance compared to the other techniques.

### Catastrophic forgetting

This is defined as incorporating new information into a plain DL model, made possible by interfering with the learned information. For instance, consider a case where there are 1000 types of flowers and a model is trained to classify these flowers, after which a new type of flower is introduced; if the model is fine-tuned only with this new class, its performance will become unsuccessful with the older classes [183, 184]. The logical data are continually collected and renewed, which is in fact a highly typical scenario in many fields, e.g. Biology. To address this issue, there is a direct solution that involves employing old and new data to train an entirely new model from scratch. This solution is time-consuming and computationally intensive; furthermore, it leads to an unstable state for the learned representation of the initial data. At this time, three different types of ML techniques, which have not catastrophic forgetting, are made available to solve the human brain problem founded on the neurophysiological theories [185, 186]. Techniques of the first type are founded on regularizations such as EWC [183] Techniques of the second type employ rehearsal training techniques and dynamic neural network architecture like iCaRL [187, 188]. Finally, techniques of the third type are founded on dual-memory learning systems [189]. Refer to [190–192] in order to gain more details.

### Model compression

To obtain well-trained models that can still be employed productively, DL models have intensive memory and computational requirements due to their huge complexity and large numbers of parameters [193, 194]. One of the fields that is characterized as data-intensive is the field of healthcare and environmental science. These needs reduce the deployment of DL in limited computational-power machines, mainly in the healthcare field. The numerous methods of assessing human health and the data heterogeneity have become far more complicated and vastly larger in size [195]; thus, the issue requires additional computation [196]. Furthermore, novel hardware-based parallel processing solutions such as FPGAs and GPUs [197–199] have been developed to solve the computation issues associated with DL. Recently, numerous techniques for compressing the DL models, designed to decrease the computational issues of the models from the starting point, have also been introduced. These techniques can be classified into four classes. In the first class, the redundant parameters (which have no significant impact on model performance) are reduced. This class, which includes the famous deep compression method, is called parameter pruning [200]. In the second class, the larger model uses its distilled knowledge to train a more compact model; thus, it is called knowledge distillation [201, 202]. In the third class, compact convolution filters are used to reduce the number of parameters [203]. In the final class, the information parameters are estimated for preservation using low-rank factorization [204]. For model compression, these classes represent the most representative techniques. In [193], it has been provided a more comprehensive discussion about the topic.

### Overfitting

DL models have excessively high possibilities of resulting in data overfitting at the training stage due to the vast number of parameters involved, which are correlated in a complex manner. Such situations reduce the model’s ability to achieve good performance on the tested data [90, 205]. This problem is not only limited to a specific field, but involves different tasks. Therefore, when proposing DL techniques, this problem should be fully considered and accurately handled. In DL, the implied bias of the training process enables the model to overcome crucial overfitting problems, as recent studies suggest [205–208]. Even so, it is still necessary to develop techniques that handle the overfitting problem. An investigation of the available DL algorithms that ease the overfitting problem can categorize them into three classes. The first class acts on both the model architecture and model parameters and includes the most familiar approaches, such as weight decay [209], batch normalization [210], and dropout [90]. In DL, the default technique is weight decay [209], which is used extensively in almost all ML algorithms as a universal regularizer. The second class works on model inputs such as data corruption and data augmentation [150, 211]. One reason for the overfitting problem is the lack of training data, which makes the learned distribution not mirror the real distribution. Data augmentation enlarges the training data. By contrast, marginalized data corruption improves the solution exclusive to augmenting the data. The final class works on the model output. A recently proposed technique penalizes the over-confident outputs for regularizing the model [178]. This technique has demonstrated the ability to regularize RNNs and CNNs.

### Vanishing gradient problem

In general, when using backpropagation and gradient-based learning techniques along with ANNs, largely in the training stage, a problem called the vanishing gradient problem arises [212–214]. More specifically, in each training iteration, every weight of the neural network is updated based on the current weight and is proportionally relative to the partial derivative of the error function. However, this weight updating may not occur in some cases due to a vanishingly small gradient, which in the worst case means that no extra training is possible and the neural network will stop completely. Conversely, similarly to other activation functions, the sigmoid function shrinks a large input space to a tiny input space. Thus, the derivative of the sigmoid function will be small due to large variation at the input that produces a small variation at the output. In a shallow network, only some layers use these activations, which is not a significant issue. While using more layers will lead the gradient to become very small in the training stage, in this case, the network works efficiently. The back-propagation technique is used to determine the gradients of the neural networks. Initially, this technique determines the network derivatives of each layer in the reverse direction, starting from the last layer and progressing back to the first layer. The next step involves multiplying the derivatives of each layer down the network in a similar manner to the first step. For instance, multiplying N small derivatives together when there are N hidden layers employs an activation function such as the sigmoid function. Hence, the gradient declines exponentially while propagating back to the first layer. More specifically, the biases and weights of the first layers cannot be updated efficiently during the training stage because the gradient is small. Moreover, this condition decreases the overall network accuracy, as these first layers are frequently critical to recognizing the essential elements of the input data. However, such a problem can be avoided through employing activation functions. These functions lack the squishing property, i.e., the ability to squish the input space to within a small space. By mapping X to max, the ReLU [91] is the most popular selection, as it does not yield a small derivative that is employed in the field. Another solution involves employing the batch normalization layer [81]. As mentioned earlier, the problem occurs once a large input space is squashed into a small space, leading to vanishing the derivative. Employing batch normalization degrades this issue by simply normalizing the input, i.e., the expression |*x*| does not accomplish the exterior boundaries of the sigmoid function. The normalization process makes the largest part of it come down in the green area, which ensures that the derivative is large enough for further actions. Furthermore, faster hardware can tackle the previous issue, e.g. that provided by GPUs. This makes standard back-propagation possible for many deeper layers of the network compared to the time required to recognize the vanishing gradient problem [215].

### Exploding gradient problem

Opposite to the vanishing problem is the one related to gradient. Specifically, large error gradients are accumulated during back-propagation [216–218]. The latter will lead to extremely significant updates to the weights of the network, meaning that the system becomes unsteady. Thus, the model will lose its ability to learn effectively. Grosso modo, moving backward in the network during back-propagation, the gradient grows exponentially by repetitively multiplying gradients. The weight values could thus become incredibly large and may overflow to become a not-a-number (NaN) value. Some potential solutions include:

1. Using different weight regularization techniques.
2. Redesigning the architecture of the network model.

### Underspecification

In 2020, a team of computer scientists at Google has identified a new challenge called underspecification [219]. ML models including DL models often show surprisingly poor behavior when they are tested in real-world applications such as computer vision, medical imaging, natural language processing, and medical genomics. The reason behind the weak performance is due to underspecification. It has been shown that small modifications can force a model towards a completely different solution as well as lead to different predictions in deployment domains. There are different techniques of addressing underspecification issue. One of them is to design “stress tests” to examine how good a model works on real-world data and to find out the possible issues. Nevertheless, this demands a reliable understanding of the process the model can work inaccurately. The team stated that “Designing stress tests that are well-matched to applied requirements, and that provide good “coverage” of potential failure modes is a major challenge”. Underspecification puts major constraints on the credibility of ML predictions and may require some reconsidering over certain applications. Since ML is linked to human by serving several applications such as medical imaging and self-driving cars, it will require proper attention to this issue.

## Applications of deep learning

**Fig. 29:**
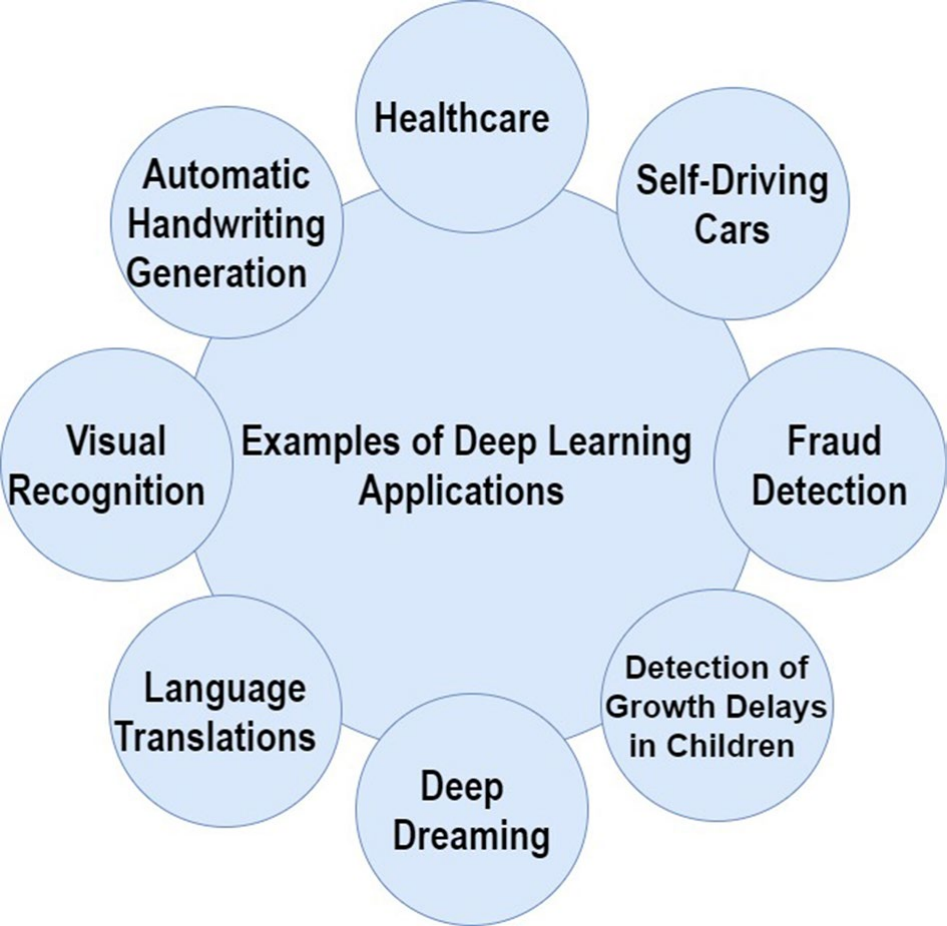
Examples of DL applications

**Fig. 30:**
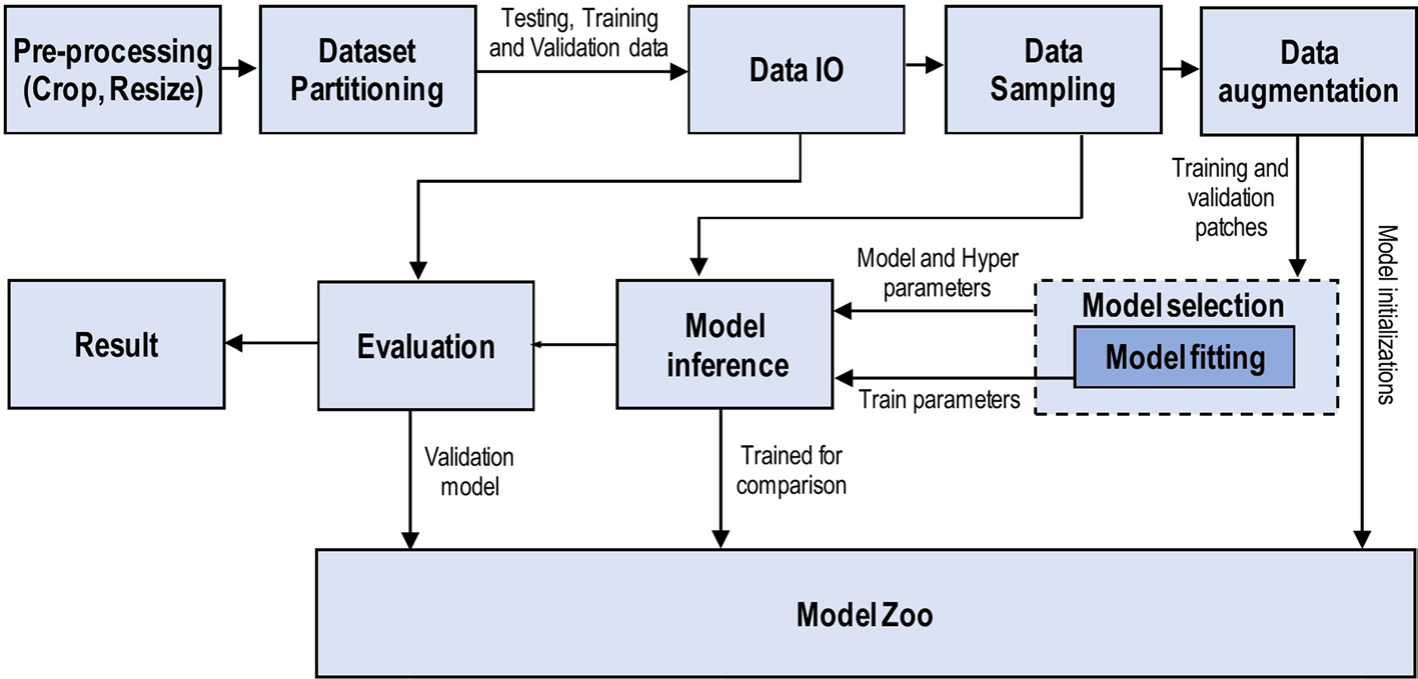
Workflow of deep learning tasks

Presently, various DL applications are widespread around the world. These applications include healthcare, social network analysis, audio and speech processing (like recognition and enhancement), visual data processing methods (such as multimedia data analysis and computer vision), and NLP (translation and sentence classification), among others (Fig. 29) [220–224]. These applications have been classified into five categories: classification, localization, detection, segmentation, and registration. Although each of these tasks has its own target, there is fundamental overlap in the pipeline implementation of these applications as shown in Fig. 30. Classification is a concept that categorizes a set of data into classes. Detection is used to locate interesting objects in an image with consideration given to the background. In detection, multiple objects, which could be from dissimilar classes, are surrounded by bounding boxes. Localization is the concept used to locate the object, which is surrounded by a single bounding box. In segmentation (semantic segmentation), the target object edges are surrounded by outlines, which also label them; moreover, fitting a single image (which could be 2D or 3D) onto another refers to registration. One of the most important and wide-ranging DL applications are in healthcare [225–230]. This area of research is critical due to its relation to human lives. Moreover, DL has shown tremendous performance in healthcare. Therefore, we take DL applications in the medical image analysis field as an example to describe the DL applications.

### Classification

Computer-Aided Diagnosis (CADx) is another title sometimes used for classification. Bharati et al. [231] used a chest X-ray dataset for detecting lung diseases based on a CNN. Another study attempted to read X-ray images by employing CNN [232]. In this modality, the comparative accessibility of these images has likely enhanced the progress of DL. [233] used an improved pre-trained GoogLeNet CNN containing more than 150,000 images for training and testing processes. This dataset was augmented from 1850 chest X-rays. The creators reorganized the image orientation into lateral and frontal views and achieved approximately 100% accuracy. This work of orientation classification has clinically limited use. As a part of an ultimately fully automated diagnosis workflow, it obtained the data augmentation and pre-trained efficiency in learning the metadata of relevant images. Chest infection, commonly referred to as pneumonia, is extremely treatable, as it is a commonly occurring health problem worldwide. Conversely, Rajpurkar et al. [234] utilized CheXNet, which is an improved version of DenseNet [112] with 121 convolution layers, for classifying fourteen types of disease. These authors used the CheXNet14 dataset [235], which comprises 112,000 images. This network achieved an excellent performance in recognizing fourteen different diseases. In particular, pneumonia classification accomplished a 0.7632 AUC score using receiver operating characteristics (ROC) analysis. In addition, the network obtained better than or equal to the performance of both a three-radiologist panel and four individual radiologists. Zuo et al. [236] have adopted CNN for candidate classification in lung nodule. Shen et al. [237] employed both Random Forest (RF) and SVM classifiers with CNNs to classify lung nodules. They employed two convolutional layers with each of the three parallel CNNs. The LIDC-IDRI (Lung Image Database Consortium) dataset, which contained 1010-labeled CT lung scans, was used to classify the two types of lung nodules (malignant and benign). Different scales of the image patches were used by every CNN to extract features, while the output feature vector was constructed using the learned features. Next, these vectors were classified into malignant or benign using either the RF classifier or SVM with radial basis function (RBF) filter. The model was robust to various noisy input levels and achieved an accuracy of 86% in nodule classification. Conversely, the model of [238] interpolates the image data missing between PET and MRI images using 3D CNNs. The Alzheimer Disease Neuroimaging Initiative (ADNI) database, containing 830 PET and MRI patient scans, was utilized in their work. The PET and MRI images are used to train the 3D CNNs, first as input and then as output. Furthermore, for patients who have no PET images, the 3D CNNs utilized the trained images to rebuild the PET images. These rebuilt images approximately fitted the actual disease recognition outcomes. However, this approach did not address the overfitting issues, which in turn restricted their technique in terms of its possible capacity for generalization. Diagnosing normal versus Alzheimer’s disease patients has been achieved by several CNN models [239, 240]. Hosseini-Asl et al. [241] attained 99% accuracy for up-to-date outcomes in diagnosing normal versus Alzheimer’s disease patients. These authors applied an auto-encoder architecture using 3D CNNs. The generic brain features were pre-trained on the CADDementia dataset. Subsequently, the outcomes of these learned features became inputs to higher layers to differentiate between patient scans of Alzheimer’s disease, mild cognitive impairment, or normal brains based on the ADNI dataset and using fine-tuned deep supervision techniques. The architectures of VGGNet and RNNs, in that order, were the basis of both VOXCNN and ResNet models developed by Korolev et al. [242]. They also discriminated between Alzheimer’s disease and normal patients using the ADNI database. Accuracy was 79% for Voxnet and 80% for ResNet. Compared to Hosseini-Asl’s work, both models achieved lower accuracies. Conversely, the implementation of the algorithms was simpler and did not require feature hand-crafting, as Korolev declared. In 2020, Mehmood et al. [240] trained a developed CNN-based network called “SCNN” with MRI images for the tasks of classification of Alzheimer’s disease. They achieved state-of-the-art results by obtaining an accuracy of 99.05%.

Recently, CNN has taken some medical imaging classification tasks to different level from traditional diagnosis to automated diagnosis with tremendous performance. Examples of these tasks are diabetic foot ulcer (DFU) (as normal and abnormal (DFU) classes) [87, 243–246], sickle cells anemia (SCA) (as normal, abnormal (SCA), and other blood components) [86, 247], breast cancer by classify hematoxylin–eosin-stained breast biopsy images into four classes: invasive carcinoma, in-situ carcinoma, benign tumor and normal tissue [42, 88, 248–252], and multi-class skin cancer classification [253–255].

In 2020, CNNs are playing a vital role in early diagnosis of the novel coronavirus (COVID-2019). CNN has become the primary tool for automatic COVID-19 diagnosis in many hospitals around the world using chest X-ray images [256–260]. More details about the classification of medical imaging applications can be found in [226, 261–265].

### Localization

Although applications in anatomy education could increase, the practicing clinician is more likely to be interested in the localization of normal anatomy. Radiological images are independently examined and described outside of human intervention, while localization could be applied in completely automatic end-to-end applications [266–268]. Zhao et al. [269] introduced a new deep learning-based approach to localize pancreatic tumor in projection X-ray images for image-guided radiation therapy without the need for fiducials. Roth et al. [270] constructed and trained a CNN using five convolutional layers to classify around 4000 transverse-axial CT images. These authors used five categories for classification: legs, pelvis, liver, lung, and neck. After data augmentation techniques were applied, they achieved an AUC score of 0.998 and the classification error rate of the model was 5.9%. For detecting the positions of the spleen, kidney, heart, and liver, Shin et al. [271] employed stacked auto-encoders on 78 contrast-improved MRI scans of the stomach area containing the kidneys or liver. Temporal and spatial domains were used to learn the hierarchal features. Based on the organs, these approaches achieved detection accuracies of 62–79%. Sirazitdinov et al. [268] presented an aggregate of two convolutional neural networks, namely RetinaNet and Mask R-CNN for pneumonia detection and localization.

### Detection

Computer-Aided Detection (CADe) is another method used for detection. For both the clinician and the patient, overlooking a lesion on a scan may have dire consequences. Thus, detection is a field of study requiring both accuracy and sensitivity [272–274]. Chouhan et al. [275] introduced an innovative deep learning framework for the detection of pneumonia by adopting the idea of transfer learning. Their approach obtained an accuracy of 96.4% with a recall of 99.62% on unseen data. In the area of COVID-19 and pulmonary disease, several convolutional neural network approaches have been proposed for automatic detection from X-ray images which showed an excellent performance [46, 276–279].

In the area of skin cancer, there several applications were introduced for the detection task [280–282]. Thurnhofer-Hemsi et al. [283] introduced a deep learning approach for skin cancer detection by fine-tuning five state-of-art convolutional neural network models. They addressed the issue of a lack of training data by adopting the ideas of transfer learning and data augmentation techniques. DenseNet201 network has shown superior results compared to other models.

Another interesting area is that of histopathological images, which are progressively digitized. Several papers have been published in this field [284–290]. Human pathologists read these images laboriously; they search for malignancy markers, such as a high index of cell proliferation, using molecular markers (e.g. Ki-67), cellular necrosis signs, abnormal cellular architecture, enlarged numbers of mitotic figures denoting augmented cell replication, and enlarged nucleus-to-cytoplasm ratios. Note that the histopathological slide may contain a huge number of cells (up to the thousands). Thus, the risk of disregarding abnormal neoplastic regions is high when wading through these cells at excessive levels of magnification. Ciresan et al. [291] employed CNNs of 11–13 layers for identifying mitotic figures. Fifty breast histology images from the MITOS dataset were used. Their technique attained recall and precision scores of 0.7 and 0.88 respectively. Sirinukunwattana et al. [292] utilized 100 histology images of colorectal adenocarcinoma to detect cell nuclei using CNNs. Roughly 30,000 nuclei were hand-labeled for training purposes. The novelty of this approach was in the use of Spatially Constrained CNN. This CNN detects the center of nuclei using the surrounding spatial context and spatial regression. Instead of this CNN, Xu et al. [293] employed a stacked sparse auto-encoder (SSAE) to identify nuclei in histological slides of breast cancer, achieving 0.83 and 0.89 recall and precision scores respectively. In this field, they showed that unsupervised learning techniques are also effectively utilized. In medical images, Albarquoni et al. [294] investigated the problem of insufficient labeling. They crowd-sourced the actual mitoses labeling in the histology images of breast cancer (from amateurs online). Solving the recurrent issue of inadequate labeling during the analysis of medical images can be achieved by feeding the crowd-sourced input labels into the CNN. This method signifies a remarkable proof-of-concept effort. In 2020, Lei et al. [285] introduced the employment of deep convolutional neural networks for automatic identification of mitotic candidates from histological sections for mitosis screening. They obtained the state-of-the-art detection results on the dataset of the International Pattern Recognition Conference (ICPR) 2012 Mitosis Detection Competition.

### Segmentation

Although MRI and CT image segmentation research includes different organs such as knee cartilage, prostate, and liver, most research work has concentrated on brain segmentation, particularly tumors [295–300]. This issue is highly significant in surgical preparation to obtain the precise tumor limits for the shortest surgical resection. During surgery, excessive sacrificing of key brain regions may lead to neurological shortfalls including cognitive damage, emotionlessness, and limb difficulty. Conventionally, medical anatomical segmentation was done by hand; more specifically, the clinician draws out lines within the complete stack of the CT or MRI volume slice by slice. Thus, it is perfect for implementing a solution that computerizes this painstaking work. Wadhwa et al. [301] presented a brief overview on brain tumor segmentation of MRI images. Akkus et al. [302] wrote a brilliant review of brain MRI segmentation that addressed the different metrics and CNN architectures employed. Moreover, they explain several competitions in detail, as well as their datasets, which included Ischemic Stroke Lesion Segmentation (ISLES), Mild Traumatic brain injury Outcome Prediction (MTOP), and Brain Tumor Segmentation (BRATS).

Chen et al. [299] proposed convolutional neural networks for precise brain tumor segmentation. The approach that they employed involves several approaches for better features learning including the DeepMedic model, a novel dual-force training scheme, a label distribution-based loss function, and Multi-Layer Perceptron-based post-processing. They conducted their method on the two most modern brain tumor segmentation datasets, i.e., BRATS 2017 and BRATS 2015 datasets. Hu et al. [300] introduced the brain tumor segmentation method by adopting a multi-cascaded convolutional neural network (MCCNN) and fully connected conditional random fields (CRFs). The achieved results were excellent compared with the state-of-the-art methods.

Moeskops et al. [303] employed three parallel-running CNNs, each of which had a 2D input patch of dissimilar size, for segmenting and classifying MRI brain images. These images, which include 35 adults and 22 pre-term infants, were classified into various tissue categories such as cerebrospinal fluid, grey matter, and white matter. Every patch concentrates on capturing various image aspects with the benefit of employing three dissimilar sizes of input patch; here, the bigger sizes incorporated the spatial features, while the lowest patch sizes concentrated on the local textures. In general, the algorithm has Dice coefficients in the range of 0.82–0.87 and achieved a satisfactory accuracy. Although 2D image slices are employed in the majority of segmentation research, Milletrate et al. [304] implemented 3D CNN for segmenting MRI prostate images. Furthermore, they used the PROMISE2012 challenge dataset, from which fifty MRI scans were used for training and thirty for testing. The U-Net architecture of Ronnerberger et al. [305] inspired their V-net. This model attained a 0.869 Dice coefficient score, the same as the winning teams in the competition. To reduce overfitting and create the model of a deeper 11-convolutional layer CNN, Pereira et al. [306] applied intentionally small-sized filters of 3x3. Their model used MRI scans of 274 gliomas (a type of brain tumor) for training. They achieved first place in the 2013 BRATS challenge, as well as second place in the BRATS challenge 2015. Havaei et al. [307] also considered gliomas using the 2013 BRATS dataset. They investigated different 2D CNN architectures. Compared to the winner of BRATS 2013, their algorithm worked better, as it required only 3 min to execute rather than 100 min. The concept of cascaded architecture formed the basis of their model. Thus, it is referred to as an InputCascadeCNN. Employing FC Conditional Random Fields (CRFs), atrous spatial pyramid pooling, and up-sampled filters were techniques introduced by Chen et al. [308]. These authors aimed to enhance the accuracy of localization and enlarge the field of view of every filter at a multi-scale. Their model, DeepLab, attained 79.7% mIOU (mean Intersection Over Union). In the PASCAL VOC-2012 image segmentation, their model obtained an excellent performance.

Recently, the Automatic segmentation of COVID-19 Lung Infection from CT Images helps to detect the development of COVID-19 infection by employing several deep learning techniques [309–312].

### Registration

Usually, given two input images, the four main stages of the canonical procedure of the image registration task are [313, 314]:

- Target Selection: it illustrates the determined input image that the second counterpart input image needs to remain accurately superimposed to.
- Feature Extraction: it computes the set of features extracted from each input image.
- Feature Matching: it allows finding similarities between the previously obtained features.
- Pose Optimization: it is aimed to minimize the distance between both input images.

Then, the result of the registration procedure is the suitable geometric transformation (e.g. translation, rotation, scaling, etc.) that provides both input images within the same coordinate system in a way the distance between them is minimal, i.e. their level of superimposition/overlapping is optimal. It is out of the scope of this work to provide an extensive review of this topic. Nevertheless, a short summary is accordingly introduced next.

Commonly, the input images for the DL-based registration approach could be in various forms, e.g. point clouds, voxel grids, and meshes. Additionally, some techniques allow as inputs the result of the Feature Extraction or Matching steps in the canonical scheme. Specifically, the outcome could be some data in a particular form as well as the result of the steps from the classical pipeline (feature vector, matching vector, and transformation). Nevertheless, with the newest DL-based methods, a novel conceptual type of ecosystem issues. It contains acquired characteristics about the target, materials, and their behavior that can be registered with the input data. Such a conceptual ecosystem is formed by a neural network and its training manner, and it could be counted as an input to the registration approach. Nevertheless, it is not an input that one might adopt in every registration situation since it corresponds to an interior data representation.

From a DL view-point, the interpretation of the conceptual design enables differentiating the input data of a registration approach into defined or non-defined models. In particular, the illustrated phases are models that depict particular spatial data (e.g. 2D or 3D) while a non-defined one is a generalization of a data set created by a learning system. Yumer et al. [315] developed a framework in which the model acquires characteristics of objects, meaning ready to identify what a more sporty car seems like or a more comfy chair is, also adjusting a 3D model to fit those characteristics while maintaining the main characteristics of the primary data. Likewise, a fundamental perspective of the unsupervised learning method introduced by Ding et al. [316] is that there is no target for the registration approach. In this instance, the network is able of placing each input point cloud in a global space, solving SLAM issues in which many point clouds have to be registered rigidly. On the other hand, Mahadevan [317] proposed the combination of two conceptual models utilizing the growth of Imagination Machines to give flexible artificial intelligence systems and relationships between the learned phases through training schemes that are not inspired on labels and classifications. Another practical application of DL, especially CNNs, to image registration is the 3D reconstruction of objects. Wang et al. [318] applied an adversarial way using CNNs to rebuild a 3D model of an object from its 2D image. The network learns many objects and orally accomplishes the registration between the image and the conceptual model. Similarly, Hermoza et al. [319] also utilize the GAN network for prognosticating the absent geometry of damaged archaeological objects, providing the reconstructed object based on a voxel grid format and a label selecting its class.

DL for medical image registration has numerous applications, which were listed by some review papers [320–322]. Yang et al. [323] implemented stacked convolutional layers as an encoder-decoder approach to predict the morphing of the input pixel into its last formation using MRI brain scans from the OASIS dataset. They employed a registration model known as Large Deformation Diffeomorphic Metric Mapping (LDDMM) and attained remarkable enhancements in computation time. Miao et al. [324] used synthetic X-ray images to train a five-layer CNN to register 3D models of a trans-esophageal probe, a hand implant, and a knee implant onto 2D X-ray images for pose estimation. They determined that their model achieved an execution time of 0.1 s, representing an important enhancement against the conventional registration techniques based on intensity; moreover, it achieved effective registrations 79–99% of the time. Li et al. [325] introduced a neural network-based approach for the non-rigid 2D–3D registration of the lateral cephalogram and the volumetric cone-beam CT (CBCT) images.

## Computational approaches

For computationally exhaustive applications, complex ML and DL approaches have rapidly been identified as the most significant techniques and are widely used in different fields. The development and enhancement of algorithms aggregated with capabilities of well-behaved computational performance and large datasets make it possible to effectively execute several applications, as earlier applications were either not possible or difficult to take into consideration.

Currently, several standard DNN configurations are available. The interconnection patterns between layers and the total number of layers represent the main differences between these configurations. The Table 2 illustrates the growth rate of the overall number of layers over time, which seems to be far faster than the “Moore’s Law growth rate”. In normal DNN, the number of layers grew by around 2.3× each year in the period from 2012 to 2016. Recent investigations of future ResNet versions reveal that the number of layers can be extended up to 1000. However, an SGD technique is employed to fit the weights (or parameters), while different optimization techniques are employed to obtain parameter updating during the DNN training process. Repetitive updates are required to enhance network accuracy in addition to a minorly augmented rate of enhancement. For example, the training process using ImageNet as a large dataset, which contains more than 14 million images, along with ResNet as a network model, take around 30K to 40K repetitions to converge to a steady solution. In addition, the overall computational load, as an upper-level prediction, may exceed 1020 FLOPS when both the training set size and the DNN complexity increase.

Prior to 2008, boosting the training to a satisfactory extent was achieved by using GPUs. Usually, days or weeks are needed for a training session, even with GPU support. By contrast, several optimization strategies were developed to reduce the extensive learning time. The computational requirements are believed to increase as the DNNs continuously enlarge in both complexity and size.

In addition to the computational load cost, the memory bandwidth and capacity have a significant effect on the entire training performance, and to a lesser extent, deduction. More specifically, the parameters are distributed through every layer of the input data, there is a sizeable amount of reused data, and the computation of several network layers exhibits an excessive computation-to-bandwidth ratio. By contrast, there are no distributed parameters, the amount of reused data is extremely small, and the additional FC layers have an extremely small computation-to-bandwidth ratio. Table 3 presents a comparison between different aspects related to the devices. In addition, the table is established to facilitate familiarity with the tradeoffs by obtaining the optimal approach for configuring a system based on either FPGA, GPU, or CPU devices. It should be noted that each has corresponding weaknesses and strengths; accordingly, there are no clear one-size-fits-all solutions.

**Table 3:** A comparison between different aspects related to the devices

Feature	Assessment	Leader
Development	CPU is the easiest to program, then GPU, then FPGA	CPU
Size	Both FPGA and CPU have smaller volume solutions due to their lower power consumption	FPGA-CPU
Customization	Broader flexibility is provided by FPGA	FPGA
Ease of change	Easier way to vary application functionality is provided by GPU and CPU	GPU-CPU
Backward compatibility	Transferring RTL to novel FPGA requires additional work. Furthermore, GPU has less stable architecture than CPU	CPU
Interfaces	Several varieties of interfaces can be implemented using FPGA	FPGA
Processing/$	FPGA configurability assists utilization in wider acceleration space. Due to the considerable processing abilities, GPU wins	FPGA-GPU
Processing/watt	Customized designs can be optimized	FPGA
Timing latency	Implemented FPGA algorithm offers deterministic timing, which is in turn much faster than GPU	FPGA
Large data analysis	FPGA performs well for inline processing, while CPU supports storage capabilities and largest memory	FPGA-CPU
DCNN inference	FPGA has lower latency and can be customized	FPGA
DCNN training	Greater float-point capabilities provided by GPU	GPU

Although GPU processing has enhanced the ability to address the computational challenges related to such networks, the maximum GPU (or CPU) performance is not achieved, and several techniques or models have turned out to be strongly linked to bandwidth. In the worst cases, the GPU efficiency is between 15 and 20% of the maximum theoretical performance. This issue is required to enlarge the memory bandwidth using high-bandwidth stacked memory. Next, different approaches based on FPGA, GPU, and CPU are accordingly detailed.

### CPU-based approach

The well-behaved performance of the CPU nodes usually assists robust network connectivity, storage abilities, and large memory. Although CPU nodes are more common-purpose than those of FPGA or GPU, they lack the ability to match them in unprocessed computation facilities, since this requires increased network ability and a larger memory capacity.

### GPU-based approach

GPUs are extremely effective for several basic DL primitives, which include greatly parallel-computing operations such as activation functions, matrix multiplication, and convolutions [326–330]. Incorporating HBM-stacked memory into the up-to-date GPU models significantly enhances the bandwidth. This enhancement allows numerous primitives to efficiently utilize all computational resources of the available GPUs. The improvement in GPU performance over CPU performance is usually 10-20:1 related to dense linear algebra operations.

Maximizing parallel processing is the base of the initial GPU programming model. For example, a GPU model may involve up to sixty-four computational units. There are four SIMD engines per each computational layer, and each SIMD has sixteen floating-point computation lanes. The peak performance is 25 TFLOPS (fp16) and 10 TFLOPS (fp32) as the percentage of the employment approaches 100%. Additional GPU performance may be achieved if the addition and multiply functions for vectors combine the inner production instructions for matching primitives related to matrix operations.

For DNN training, the GPU is usually considered to be an optimized design, while for inference operations, it may also offer considerable performance improvements.

### FPGA-based approach

FPGA is wildly utilized in various tasks including deep learning [199, 247, 331–334]. Inference accelerators are commonly implemented utilizing FPGA. The FPGA can be effectively configured to reduce the unnecessary or overhead functions involved in GPU systems. Compared to GPU, the FPGA is restricted to both weak-behaved floating-point performance and integer inference. The main FPGA aspect is the capability to dynamically reconfigure the array characteristics (at run-time), as well as the capability to configure the array by means of effective design with little or no overhead.

As mentioned earlier, the FPGA offers both performance and latency for every watt it gains over GPU and CPU in DL inference operations. Implementation of custom high-performance hardware, pruned networks, and reduced arithmetic precision are three factors that enable the FPGA to implement DL algorithms and to achieve FPGA with this level of efficiency. In addition, FPGA may be employed to implement CNN overlay engines with over 80% efficiency, eight-bit accuracy, and over 15 TOPs peak performance; this is used for a few conventional CNNs, as Xillinx and partners demonstrated recently. By contrast, pruning techniques are mostly employed in the LSTM context. The sizes of the models can be efficiently minimized by up to 20×, which provides an important benefit during the implementation of the optimal solution, as MLP neural processing demonstrated. A recent study in the field of implementing fixed-point precision and custom floating-point has revealed that lowering the 8-bit is extremely promising; moreover, it aids in supplying additional advancements to implementing peak performance FPGA related to the DNN models.

## Evaluation metrics

Evaluation metrics adopted within DL tasks play a crucial role in achieving the optimized classifier [335]. They are utilized within a usual data classification procedure through two main stages: training and testing. It is utilized to optimize the classification algorithm during the training stage. This means that the evaluation metric is utilized to discriminate and select the optimized solution, e.g., as a discriminator, which can generate an extra-accurate forecast of upcoming evaluations related to a specific classifier. For the time being, the evaluation metric is utilized to measure the efficiency of the created classifier, e.g. as an evaluator, within the model testing stage using hidden data. As given in Eq. 20, TN and TP are defined as the number of negative and positive instances, respectively, which are successfully classified. In addition, FN and FP are defined as the number of misclassified positive and negative instances respectively. Next, some of the most well-known evaluation metrics are listed below.

1. Accuracy: Calculates the ratio of correct predicted classes to the total number of samples evaluated (Eq. 20).
  20 $$ Accuracy = \frac{TP+TN }{TP+TN+FP+FN} $$
2. Sensitivity or Recall: Utilized to calculate the fraction of positive patterns that are correctly classified (Eq. 21).
  21 $$ Sensitivity=\frac{TP}{TP+FN }$$
3. Specificity: Utilized to calculate the fraction of negative patterns that are correctly classified (Eq. 22).
  22 $$ Specificity =\frac{TN }{FP+TN }$$
4. Precision: Utilized to calculate the positive patterns that are correctly predicted by all predicted patterns in a positive class (Eq. 23).
  23 $$ Precision=\frac{TP }{TP+FP} $$
5. F1-Score: Calculates the harmonic average between recall and precision rates (Eq. 24).
  24 $$ F1_{score} = 2\times \frac{Precision\times Recall}{Precision+Recall} $$
6. J Score: This metric is also called Youdens J statistic. Eq. 25 represents the metric.
  25 $$ J_{score} = Sensitivity + Specificity -1 $$
7. False Positive Rate (FPR): This metric refers to the possibility of a false alarm ratio as calculated in Eq. 26
  26 $$ FPR = 1- Specificity$$
8. Area Under the ROC Curve: AUC is a common ranking type metric. It is utilized to conduct comparisons between learning algorithms [336–338], as well as to construct an optimal learning model [339, 340]. In contrast to probability and threshold metrics, the AUC value exposes the entire classifier ranking performance. The following formula is used to calculate the AUC value for two-class problem [341] (Eq. 27)
  27 $$ AUC = \frac{S_{p}-n_{p} (n_{n}+1)/2}{n_{p}n_{n}} $$
  Here, $S_{p}$ represents the sum of all positive ranked samples. The number of negative and positive samples is denoted as $n_{n}$ and $n_{p}$, respectively. Compared to the accuracy metrics, the AUC value was verified empirically and theoretically, making it very helpful for identifying an optimized solution and evaluating the classifier performance through classification training.
  When considering the discrimination and evaluation processes, the AUC performance was brilliant. However, for multiclass issues, the AUC computation is primarily cost-effective when discriminating a large number of created solutions. In addition, the time complexity for computing the AUC is $O \left( |C|^{2} \; n\log n\right)$ with respect to the Hand and Till AUC model [341] and $O \left( |C| \; n\log n\right)$ according to Provost and Domingo’s AUC model [336].

## Frameworks and datasets

Several DL frameworks and datasets have been developed in the last few years. various frameworks and libraries have also been used in order to expedite the work with good results. Through their use, the training process has become easier. Table 4 lists the most utilized frameworks and libraries.

**Table 4:** List of the most common frameworks and libraries

Framework	License	Core language	Year of release	Homepages
TensorFlow	Apache 2.0	C++ & Python	2015	https://www.tensorflow.org/
Keras	MIT	Python	2015	https://keras.io/
Caffe	BSD	C++	2015	http://caffe.berkeleyvision.org/
MatConvNet	Oxford	MATLAB	2014	http://www.vlfeat.org/matconvnet/
MXNet	Apache 2.0	C++	2015	https://github.com/dmlc/mxnet
CNTK	MIT	C++	2016	https://github.com/Microsoft/CNTK
Theano	BSD	Python	2008	http://deeplearning.net/software/theano/
Torch	BSD	C & Lua	2002	http://torch.ch/
DL4j	Apache 2.0	Java	2014	https://deeplearning4j.org/
Gluon	AWS Microsoft	C++	2017	https://github.com/gluon-api/gluon-api/
OpenDeep	MIT	Python	2017	http://www.opendeep.org/

Based on the star ratings on Github, as well as our own background in the field, TensorFlow is deemed the most effective and easy to use. It has the ability to work on several platforms. (Github is one of the biggest software hosting sites, while Github stars refer to how well-regarded a project is on the site). Moreover, there are several other benchmark datasets employed for different DL tasks. Some of these are listed in Table 5.

**Table 5:** Benchmark datasets

Dataset	Num. of classes	Applications	Link to dataset
ImageNet	1000	Image classification, object localization, object detection, etc.	http://www.image-net.org/
CIFAR10/100	10/100	Image classification	https://www.cs.toronto.edu/~kriz/cifar.html
MNIST	10	Classification of handwritten digits	http://yann.lecun.com/exdb/mnist/
Pascal VOC	20	Image classification, segmentation, object detection	http://host.robots.ox.ac.uk/pascal/VOC/voc2012/
Microsoft COCO	80	Object detection, semantic segmentation	https://cocodataset.org/#home
YFCC100M	8M	Video and image understanding	http://projects.dfki.unikl.de/yfcc100m/
YouTube-8M	4716	Video classification	https://research.google.com/youtube8m/
UCF-101	101	Human action detection	https://www.crcv.ucf.edu/data/UCF101.php
Kinetics	400	Human action detection	https://deepmind.com/research/open-source/kinetics
Google Open Images	350	Image classification, segmentation, object detection	https://storage.googleapis.com/openimages/web/index.html
CalTech101	101	Classification	http://www.vision.caltech.edu/Image_Datasets/Caltech101/
Labeled Faces in the Wild	–	Face recognition	http://vis-www.cs.umass.edu/lfw/
MIT-67 scene dataset	67	Indoor scene recognition	http://web.mit.edu/torralba/www/indoor.htm

## Summary and conclusion

Finally, it is mandatory the inclusion of a brief discussion by gathering all the relevant data provided along this extensive research. Next, an itemized analysis is presented in order to conclude our review and exhibit the future directions.

- DL already experiences difficulties in simultaneously modeling multi-complex modalities of data. In recent DL developments, another common approach is that of multimodal DL.
- DL requires sizeable datasets (labeled data preferred) to predict unseen data and to train the models. This challenge turns out to be particularly difficult when real-time data processing is required or when the provided datasets are limited (such as in the case of healthcare data). To alleviate this issue, TL and data augmentation have been researched over the last few years.
- Although ML slowly transitions to semi-supervised and unsupervised learning to manage practical data without the need for manual human labeling, many of the current deep-learning models utilize supervised learning.
- The CNN performance is greatly influenced by hyper-parameter selection. Any small change in the hyper-parameter values will affect the general CNN performance. Therefore, careful parameter selection is an extremely significant issue that should be considered during optimization scheme development.
- Impressive and robust hardware resources like GPUs are required for effective CNN training. Moreover, they are also required for exploring the efficiency of using CNN in smart and embedded systems.
- In the CNN context, ensemble learning [342, 343] represents a prospective research area. The collection of different and multiple architectures will support the model in improving its generalizability across different image categories through extracting several levels of semantic image representation. Similarly, ideas such as new activation functions, dropout, and batch normalization also merit further investigation.
- The exploitation of depth and different structural adaptations is significantly improved in the CNN learning capacity. Substituting the traditional layer configuration with blocks results in significant advances in CNN performance, as has been shown in the recent literature. Currently, developing novel and efficient block architectures is the main trend in new research models of CNN architectures. HRNet is only one example that shows there are always ways to improve the architecture.
- It is expected that cloud-based platforms will play an essential role in the future development of computational DL applications. Utilizing cloud computing offers a solution to handling the enormous amount of data. It also helps to increase efficiency and reduce costs. Furthermore, it offers the flexibility to train DL architectures.
- With the recent development in computational tools including a chip for neural networks and a mobile GPU, we will see more DL applications on mobile devices. It will be easier for users to use DL.
- Regarding the issue of lack of training data, It is expected that various techniques of transfer learning will be considered such as training the DL model on large unlabeled image datasets and next transferring the knowledge to train the DL model on a small number of labeled images for the same task.
- Last, this overview provides a starting point for the community of DL being interested in the field of DL. Furthermore, researchers would be allowed to decide the more suitable direction of work to be taken in order to provide more accurate alternatives to the field.

## Data Availability

Not applicable.
